# Astrocytes Coordinate Neurovascular Coupling During Prolonged Sensory Demand via AQP4/TRPA1/BEST1/D‐Serine Signaling

**DOI:** 10.1002/advs.77878

**Published:** 2026-09-29

**Authors:** Kayoung Han, Minwoo Wendy Jang, Taeyoung Park, Ki Jung Kim, Victor James Drew, Sunpil Kim, Tai Young Kim, Mingu Gordon Park, Myeongju Kim, Seung Won Chung, Minah Suh, C. Justin Lee

**Affiliations:** ^1^ Center for Neuroscience Imaging Research Institute for Basic Science (IBS) Suwon Republic of Korea; ^2^ Biomedical Institute for Convergence at SKKU (BICS) Sungkyunkwan University Suwon Republic of Korea; ^3^ Center for Memory and Glioscience Institute for Basic Science (IBS) Daejeon Republic of Korea; ^4^ Department of Biomedical Engineering Sungkyunkwan University Suwon Republic of Korea; ^5^ Department of Intelligent Precision Healthcare Convergence (IPHC) Sungkyunkwan University Suwon Republic of Korea

**Keywords:** aquaporin‐4, astrocyte, cerebral blood volume, D‐serine, Gliotransmission, neurovascular coupling, sensory stimulation, transient receptor potential channel, vascular tone

## Abstract

Astrocytes are recognized as key components of neurovascular coupling (NVC), yet whether and how astrocytic engagement in NVC depends on sensory stimulus duration remains unclear. Here, we investigated a duration‐dependent astrocytic mechanism underlying cerebral blood volume (CBV) modulation by comparing neurovascular responses to brief (5 s) and prolonged (20 s) sensory stimulation, using in vivo intrinsic optical signal (IOS) imaging and two‐photon microscopy combined with electrophysiology and vascular cannulation. We show that prolonged stimulation selectively recruits an integrated astrocytic signaling cascade involving aquaporin‐4 (AQP4)‐mediated volume changes, Ca^2+^ influx through mechanosensitive transient receptor potential ankyrin 1 (TRPA1), and D‐serine release through the Ca^2+^‐dependent Bestrophin‐1 (BEST1) channel. This signaling cascade drives sustained neuronal activity, leading to CBV modulation and sensory perception. Genetic ablation of each component in this pathway selectively impairs CBV responses, neuronal activity, and sensory behavior during prolonged stimulation, while exogenous D‐serine fully restores these deficits. We further demonstrate that tonic astrocytic D‐serine release via BEST1 directly regulates the basal tone of penetrating arterioles. These findings together identify astrocytic D‐serine as one of the key gliotransmitters that modulate CBV and neuronal activity during prolonged sensory demand and fine‐tune vascular tone in resting states.

## Graphical abstract

Neuronal activation leads to astrocytic transient volume changes, activating transient receptor potential ankyrin 1 (TRPA1) channels, leading to Ca^2+^ influx. Activation of TRPA1 causes the release of D‐serine through BEST1. Finally, the augmented extracellular levels of D‐serine boost neuronal activity by binding GluN1‐containing NMDARs, leading to CBV changes.

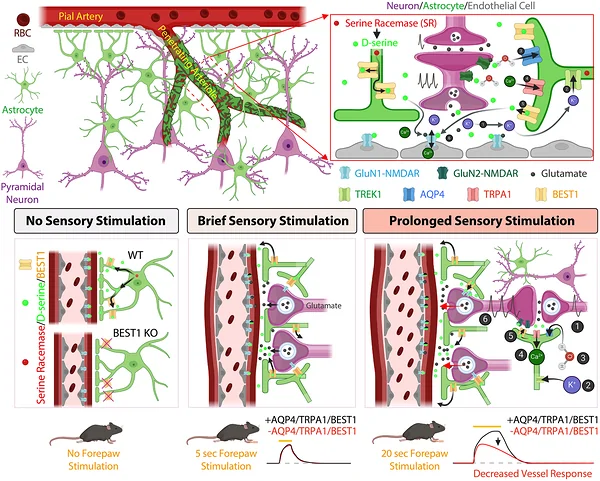

## 1 Introduction

The brain constantly regulates cerebral blood flow (CBF) to supply metabolites such as glucose and oxygen [1] in response to changing neuronal demands, an adaptive process called functional hyperemia or neurovascular coupling (NVC). These dynamics are achieved via an integrated action of neurons, astrocytes, mural cells, endothelial cells, and blood vessels forming neurovascular units (NVUs). Emerging evidence suggests that neuronal activation upon sensory stimulation increases both CBF and astrocytic calcium (Ca^2^^+^) levels [2], thereby positioning astrocytes as critical intermediaries between synaptic activity and vascular regulation.

Activated presynaptic neurons release neurotransmitters, such as glutamate, which act on postsynaptic receptors as well as astrocytic metabotropic glutamate receptors (mGluRs), leading to astrocytic Ca^2+^ elevations. These Ca^2+^ signals have been linked to the release of vasoactive mediators, including cyclooxygenase (COX)‐derived prostaglandin E2 (PGE2), arachidonic acid (AA) metabolites EET and 20‐HEPE, as well as ATP, nitric oxide (NO), and K^+^ [2, 3, 4, 5, 6, 7], which collectively regulate vascular responses. In parallel, postsynaptic activation induces neuronal depolarization through AMPA and NMDA receptors, followed by NO release via neuronal nitric oxide synthase (nNOS) [8] and K^+^ efflux during membrane repolarization. The released K^+^ in the extracellular space is taken up by astrocytes, affecting astrocytic volume changes and increasing intracellular Ca^2+^ and related downstream signaling [9, 10]. Together, these neuron‐astrocyte interactions highlight the complexity of astrocytic contribution to NVC.

Indeed, the involvement of astrocytes in NVC is still debated, as their contribution appears to depend on experimental conditions such as stimulus duration [11, 12, 13], vascular compartment [4, 14, 15, 16], neuromodulator, or behavioral state [17, 18]. A recent study has shown that astrocytic Ca^2^^+^ elevations contribute to vascular dilation during sustained neuronal activation, suggesting that astrocyte participation in NVC may depend on the duration of neuronal activity [12]. Nevertheless, the mechanisms underlying the preferential recruitment of astrocytes during prolonged stimulation and how astrocytes are engaged in a stimulus‐duration‐dependent manner remain unknown.

Astrocytic volume dynamics represent a potential mechanism by which astrocytes may sense and integrate sustained neuronal activity. Aquaporin‐4 (AQP4), primarily expressed in the astrocytic endfeet and processes, serves as a water channel [10] and regulates astrocytic volume changes necessary to maintain osmotic equilibrium [19]. AQP4 is involved in the initiation of the astrocytic swelling process in the hippocampus, as evidenced by the near‐complete elimination of brain IOS signals and the reduction in astrocytic volume changes following AQP4 gene silencing [20, 21]. Its proximity to synapses and blood vessels allows AQP4‐induced changes in the extracellular space to influence neuronal excitability [22, 23], synaptic plasticity [24], brain plasticity [19], learning and memory [19], and blood oxygen level‐dependent (BOLD) signal [25]. These changes can activate swelling‐activated mechanosensitive ion channels, such as transient receptor potential (TRP) channels [10]. Among various TRP channels, TRPA1 is responsible for Ca^2+^ influx, independent of astrocyte IP_3_R signaling [10, 26], and is expressed in astrocytic cell bodies, processes, and leaflets [27, 28, 29]. Our previous research has shown that AQP4‐driven increases in astrocytic volume result in an intracellular Ca^2+^ rise through TRPA1, as indicated by reduced Ca^2+^ responses when incubated with a selective TRPA1 blocker, HC030031 [10]. Notably, AQP4 gene silencing significantly attenuated astrocytic Ca^2^^+^ rise in the hippocampus [21], supporting a functional association between AQP4 and TRPA1. Furthermore, we have reported that low‐intensity, low‐frequency ultrasound (LILFU), a form of mechanical stimulation delivered through sound waves, causes TRPA1 channel activation, leading to neuronal NMDAR activation through the release of a gliotransmitter via the Ca^2+^‐dependent BEST1 channel [30]. Among various gliotransmitters, D‐serine acts as a co‐agonist for the NMDA receptor and is extensively distributed in astrocytes [31, 32]. D‐serine has been reported to be released as a gliotransmitter through Ca^2+^‐dependent opening of the BEST1 channel [30], contributing to astrocyte‐neuron communication [30, 33, 34]. In addition, although D‐serine is not a classical vasodilator, D‐serine has been implicated in triggering vessel dilations by acting on endothelial NMDARs [35, 36]. Astrocyte Ca^2+^ uncaging leads to D‐serine‐dependent vasodilatory responses independent of tetrodotoxin (TTX) pretreatment, suggesting that neuronal excitation is not necessary for direct astrocyte‐mediated vasodilation [35]. Moreover, glutamate‐ and D‐serine‐induced vasodilation is inhibited after denuding the endothelium and by selective inhibition or genetic knockout of endothelial nitric oxide synthase (eNOS), implying endothelial‐dependent processes in vasodilation [36]. Endothelial GluN1 gene silencing attenuates astrocyte‐induced NO production and vasodilation [37], further suggesting a mechanism in which astrocyte‐derived D‐serine promotes endothelial NMDAR‐dependent vasodilation. Collectively, these results imply the possible contributions of astrocytic AQP4/TRPA1/BEST1/D‐serine signaling in NVC.

Despite these insights, whether and how astrocytic AQP4/TRPA1/BEST1/D‐serine signaling contributes to NVC in vivo remains unknown. In particular, it is unclear whether this pathway underlies the selective engagement of astrocytes during prolonged sensory stimulation and whether astrocytic signaling also regulates vascular tone in the absence of neuronal activation. In this study, we address these questions by systematically comparing neurovascular responses to brief and prolonged sensory stimulation. We identify a stimulation duration‐dependent astrocytic signaling pathway that couples sustained neuronal activity to cerebral blood volume regulation and sensory perception, and further reveal a role of tonic astrocytic D‐serine release in controlling basal vascular tone.

## 2 Results

### 2.1 Astrocytic AQP4 Channel Contributes to CBV, Neuronal Activity, and Sensory Perception

To investigate whether the astrocytic AQP4 water channel influences CBV depending on the duration of sensory stimulation in the primary somatosensory cortex, we measured CBV changes using IOS imaging, which provides a well‐organized topographical representation of the pial surface [38]. First, we conducted a genetic ablation of AQP4 by administering intracortical injections of AAV (AAV‐*Aqp4* shRNA‐mCherry) [10, 39] into the primary somatosensory cortex of the forelimb (S1FL) region (Figure 1a). After a 2‐week recovery period, we confirmed successful astrocytic AQP4 gene silencing compared to the control virus (AAV‐scrambled‐mCherry) (Figure S1a,b), with a modest increase in GFAP immunoreactivity, suggesting that AQP4 gene silencing was associated with minimal astrogliosis without evidence of significant neuroinflammation (Figure S2). Given our hypothesis that the astrocytic regulatory role in NVC may depend on the duration of sensory stimulation, we employed two stimulation parameters: brief (5 s) and prolonged (20 s) stimulations for this study. Mice were anesthetized with urethane (1.25 g/kg) during the experiments, a widely used anesthetic that provides long‐lasting anesthesia with relatively stable physiological conditions while preserving neuronal and vascular function [40, 41, 42, 43]. Changes in total hemoglobin (HbT), reflecting CBV changes in both pial arteries and penetrating arterioles, were measured using an sCMOS digital camera with a 546 ± 30 nm bandpass filter, following brief or prolonged 0.5 mA electrical forepaw stimulation to evoke somatosensory‐driven neurovascular responses without prominent nociceptive activation under physiologically stable conditions (Figure 1b, Figures S3 and S4). After CBV measurements, an electrode for local field potential (LFP) recording was inserted into cortical layers II/III of the central area of the activated region in response to forepaw electrical stimulation (Figure 1b). We also confirmed the expression of the *Aqp4* shRNA virus around cerebral blood vessels in the S1FL region using in vivo two‐photon microscopic imaging (Figure 1c).

**FIGURE 1:**
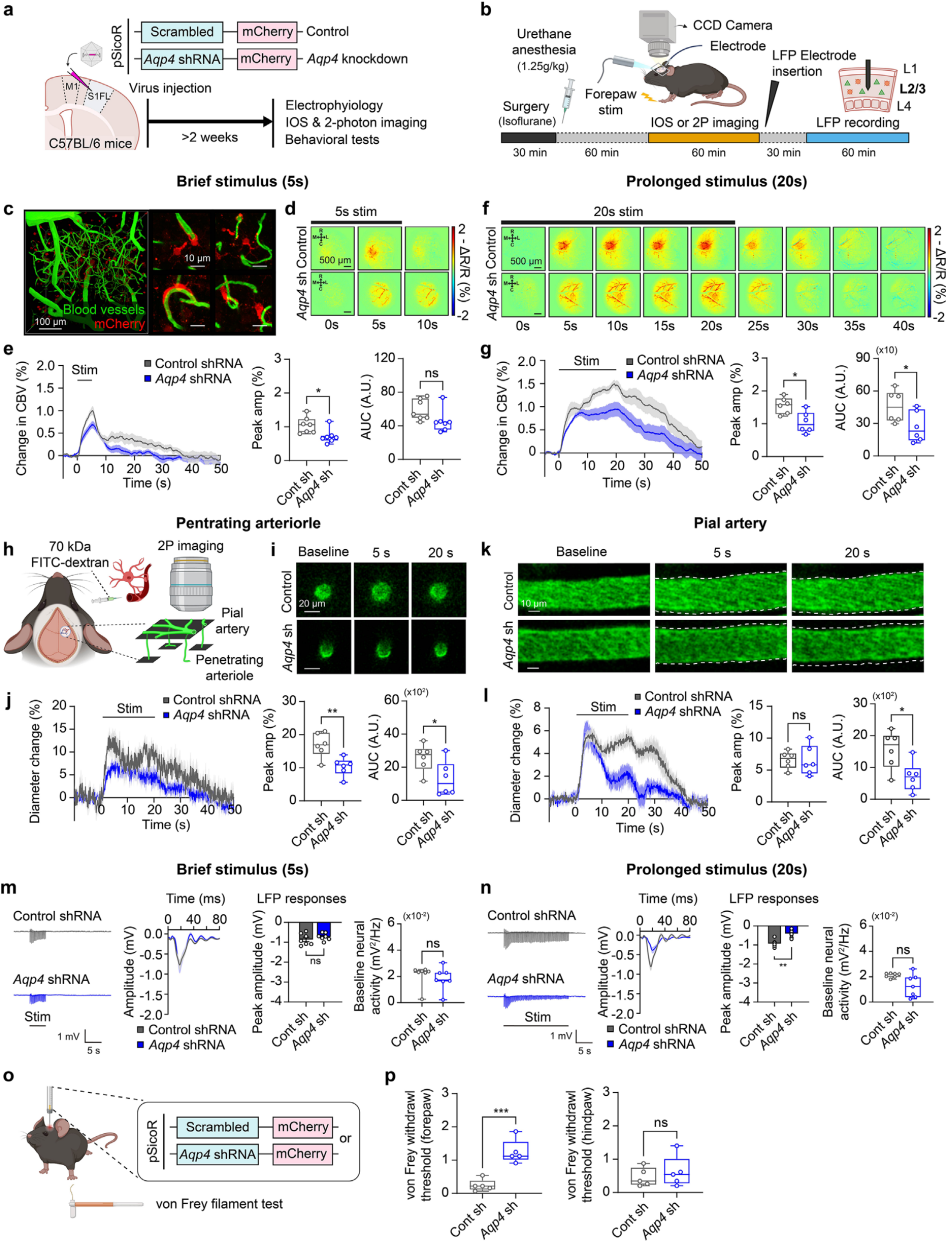
Effects of AQP4 knockdown on sensory‐evoked neural activities, CBV, and sensory perception. (a) Schematic illustrations of injecting pSicoR AAV virus carrying control shRNA or *Aqp4* shRNA into the mouse S1FL and a timeline of the experiments. (b) Experimental procedures for stereotaxic surgery, in vivo two‐photon (2P) imaging, local field potential (LFP) recording, and intrinsic optical signal (IOS) imaging. (c) Representative in vivo two‐photon images of blood vessels and AAV‐*Aqp4* shRNA‐mCherry in the S1FL region. (d) Representative spatiotemporal IOS images following brief stimulation in control mice and AQP4 knockdown mice. (e) Representative trace of CBV changes and average peak amplitude (%) and area under the curve of HbT signal following brief stimulation in control mice and AQP4 knockdown mice (Control shRNA; *n*  = 7, *Aqp4* shRNA; *n*  = 7, Peak amplitude; ^*^ *p* = 0.019, AUC; *p* = 0.074, ns, not significant, Unpaired t‐test). (f) Representative spatiotemporal IOS images following prolonged stimulation in control mice and AQP4 knockdown mice. (g) Representative trace of CBV changes and average peak amplitude (%) and area under the curve of HbT signal following prolonged stimulation in control mice and AQP4 knockdown mice (Control shRNA; *n*  = 6, *Aqp4* shRNA; *n*  = 6, Peak amplitude; ^*^ *p* = 0.011, AUC; ^*^ *p* = 0.048, Unpaired t‐test). (h) Schematic illustrations of in vivo two‐photon imaging of a pial artery and a penetrating arteriole. (i) Representative images of penetrating arteriole diameter changes in control mice and AQP4 knockdown mice. (j) Representative trace of diameter changes, average peak amplitude (%), and area under the curve of a penetrating arteriole in control mice and AQP4 knockdown mice (Control shRNA; *n*  = 6, *Aqp4* shRNA; *n*  = 6, Peak amplitude; ^**^ *p* = 0.0062, AUC; ^*^ *p* = 0.043, Unpaired t‐test). (k) Representative images of pial artery diameter changes in control mice and AQP4 knockdown mice. (l) Representative trace of diameter changes, average the peak amplitude (%), and area under the curve of a pial arteriole in control mice and AQP4 knockdown mice (Control shRNA; *n*  = 6, *Aqp4* shRNA; *n*  = 6, Peak amplitude; *p* = 0.92, AUC; ^*^ *p* = 0.019, ns, not significant, Unpaired t‐test). (m) Representative LFP traces, amplitudes, and peak amplitude during brief stimulation and basal neuronal activity before brief stimulation (Control shRNA; *n*  = 7, *Aqp4* shRNA; *n*  = 7, Peak amplitude; *p* = 0.090, Baseline activity; *p* = 0.57, ns, not significant, Unpaired t‐test). (n) Representative LFP traces, amplitudes, and peak amplitude during prolonged stimulation and basal neuronal activity before brief stimulation (Control shRNA; *n*  = 7, *Aqp4* shRNA; *n*  = 7, Peak amplitude; ^***^ *p* = 0.00035, Baseline activity; *p* = 0.57, ns, not significant, Unpaired t‐test). (o) Illustration of the von Frey filament test. (p) Average von Frey withdrawal threshold in forepaw stimulation and hindpaw stimulation (Control shRNA; *n*  = 5, *Aqp4* shRNA; *n*  = 5, Forepaw; ^***^ *p* = 0.00051, Hindpaw; *p* = 0.53, ns, not significant, Unpaired t‐test).

To elucidate the role of the AQP4 channel on CBV, we measured CBV following sensory stimulation in AQP4 knockdown and control mice. We found that electrical forepaw stimulation increased CBV in the S1FL region (Figure 1d,f). Notably, following brief stimulation, AQP4 knockdown reduced peak amplitude compared to control mice (Figure 1e). However, the time of onset and time to peak of CBV changes remained intact in AQP4 knockdown mice (Figure S5a). Prolonged stimulation also significantly diminished CBV in AQP4 knockdown mice compared to control mice, with a significant reduction in peak amplitude and area under the curve (AUC) (Figure 1g). The time of onset and time to half maximum of CBV changes during prolonged stimulation showed no differences between AQP4 knockdown and control mice (Figure S5a).

It has been previously reported that pial arteries have no connection with astrocytic endfeet, while penetrating arterioles are enwrapped by astrocytic endfeet [44]. Therefore, we explored the differential effects of AQP4 knockdown on pial arteries and penetrating arterioles by measuring their diameter changes upon sensory stimulation. To visualize diameter changes in penetrating arterioles and pial arteries, we injected 5% 70 kDa FITC‐dextran via the retro‐orbital sinus (Figure 1h). Consistent with the CBV results, AQP4 knockdown significantly reduced the peak amplitude and AUC of penetrating arteriole dilation and delayed its onset (Figure 1i,j, Figure S5b). In pial arteries, AQP4 knockdown did not affect to the peak amplitude, but significantly reduced the AUC and FWHM of the dilation response, indicating a shorter and less sustained vascular response (Figure 1k,l, Figure S5b). These results indicate that astrocytic AQP4 plays a crucial role in the magnitude and sustainability of CBV, possibly through astrocytic volume changes induced by sensory‐evoked neuronal activation in S1FL.

We then sought to investigate whether astrocytic AQP4 channel impacts neuronal activity upon sensory stimulation by recording LFP in layer II/III of S1FL. First of all, AQP4 knockdown did not affect basal neuronal activity (Figure 1m,n). More importantly, brief stimulation did not alter neuronal activity in the AQP4 knockdown mice compared to the control mice (Figure 1m), validating that the sensory stimulation can initiate neuronal activation independent of AQP4. However, sustained neuronal activity in response to prolonged stimulation was not maintained in the absence of AQP4 (Figure 1n). These results indicate that astrocytic AQP4 controls neuronal activity during prolonged sensory stimulation.

We next investigated whether astrocytic AQP4 impacts sensory perception in awake mice by measuring mechanical sensitivity using the von Frey test (Figure 1o). We did not observe any significant difference in the withdrawal threshold following hindpaw stimulation (Figure 1p) and basal locomotion activity (Figure S6a,b) when AQP4 was knocked down in S1FL of both hemispheres. However, we found that the withdrawal threshold was significantly increased following forepaw stimulation in AQP4 knockdown mice (Figure 1p). In parallel, we induced unilateral AQP4 gene silencing and confirmed that the forepaw withdrawal threshold increased only on the contralateral side of the *Aqp4* shRNA injection (Figure S7), demonstrating the functional role of AQP4 in sensory responses. These results indicate that astrocytic AQP4 is necessary for the control of sensory perception.

### 2.2 Astrocytic AQP4 Regulates Activity‐Dependent Volume Responses and Ca^2+^ Signaling

We next investigated how astrocytic AQP4 may contribute to sensory‐evoked neural and vascular responses. In our previous work in the hippocampus, AQP4 silencing markedly suppressed IOS responses and reduced single‐astrocyte volume changes, supporting an important role for AQP4 in astrocyte swelling [20]. To determine whether AQP4 similarly contributes to activity‐dependent volume and Ca^2+^ responses in the S1FL, we performed quantitative IOS measurements and astrocytic Ca^2+^ imaging in acute brain slices subjected to brief (5 s) and prolonged stimulation (20 s).

For AQP4 knockdown and astrocytic Ca^2+^ imaging, AAV expressing *Aqp4* shRNA and gfaABC1D‐Lck‐GCaMP6f was co‐injected into the S1FL (Figure 2a). After validating different electrical stimulation protocols, we selected stimulation of 10 Hz and an intensity of 100 µA, which induced robust IOS responses under both stimulation conditions (Figure 2b). We found that AQP4 gene silencing resulted in significantly reduced peak and AUC of the IOS responses following brief and prolonged stimulation (Figure 2c,d). Although the effect in the S1FL was less pronounced than the near‐complete suppression previously observed in the hippocampus, AQP4 silencing significantly attenuated astrocyte volume changes by approximately 60–70% (Figure 2c,d). These findings demonstrate that AQP4 is required for activity‐dependent astrocyte volume responses in the S1FL.

**FIGURE 2:**
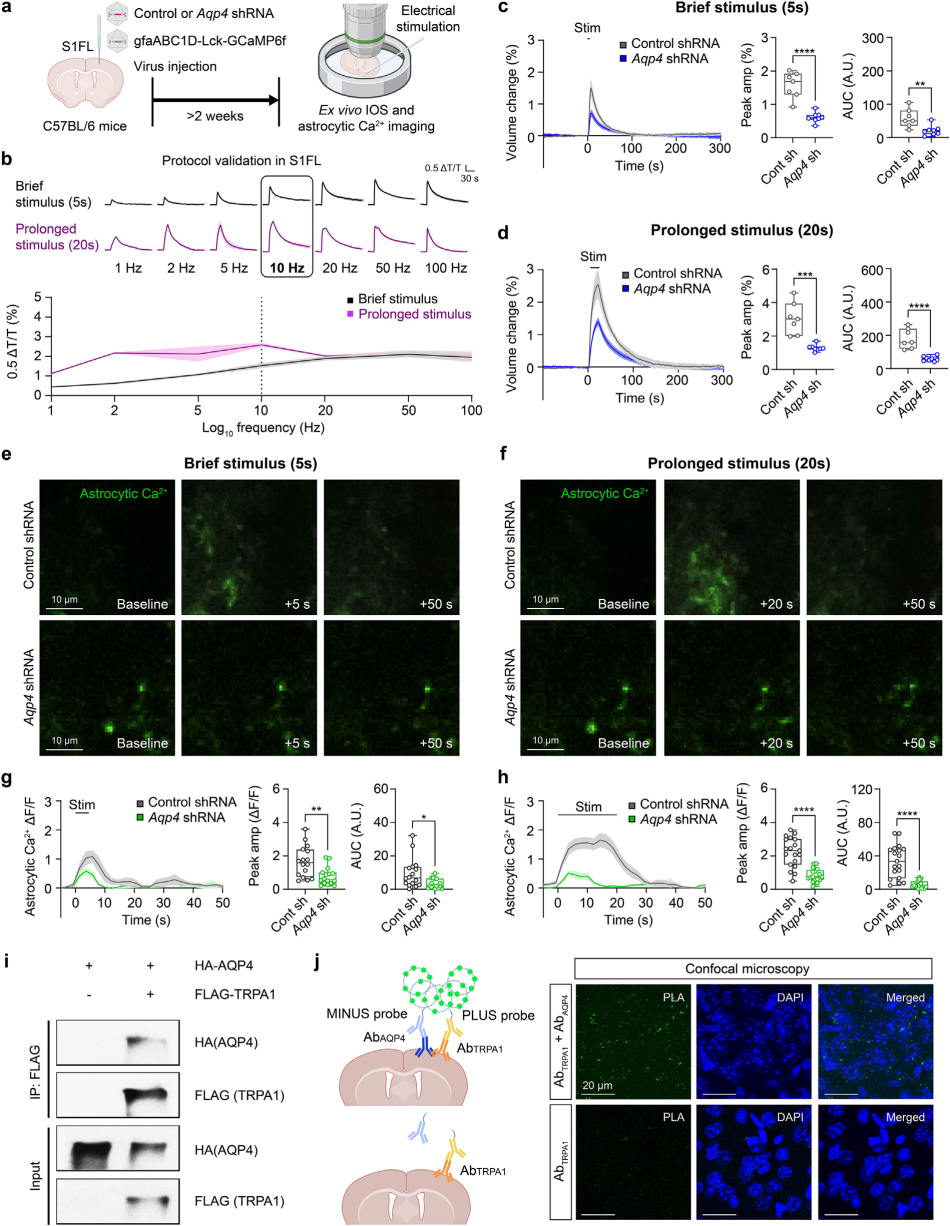
Effects of AQP4 knockdown on stimulation‐evoked volume change and astrocytic Ca^2+^ responses and its association with TRPA1. (a) Schematic diagram showing unilateral virus injection into the cortical S1FL region and ex vivo IOS in the brain slice of S1FL. (b) Representative traces of IOS responses by brief and prolonged stimulation with various conditions and a summary graph of amplitude (*n* = 3–7). (c, d) Representative traces and average bar graphs of peak and AUC of IOS following brief (5s) and prolonged stimulation (20s) in control shRNA and AQP4 knockdown mice (Control shRNA; *n* = 7, *Aqp4* shRNA; *n* = 8, Peak amplitude; ^****^ *p* < 0.0001, AUC; ^**^ *p* = 0.0054 for brief stimulation; Peak amplitude; ^***^ *p* = 0.0002, AUC; ^****^ *p* < 0.0001 for prolonged stimulation, Unpaired t‐test). (e) Representative images of astrocytic Ca^2^^+^ activity following brief and prolonged stimulation in control shRNA and AQP4 knockdown mice. (f) Average time‐course traces of stimulation‐evoked astrocytic Ca^2^^+^ responses during brief stimulation and quantification of the peak amplitude and AUC (Control shRNA; *n* = 16, *Aqp4* shRNA; *n* = 16, Peak amplitude; ^**^ *p* = 0.0026, AUC; ^*^ *p* = 0.0125 for brief stimulation; Control shRNA; *n* = 19, *Aqp4* shRNA; *n* = 17, Peak amplitude; ^****^ *p* < 0.0001, AUC; ^****^ *p* < 0.0001 for prolonged stimulation, Unpaired t‐test). (i) Co‐IP data showing that AQP4 (HA) is immunoprecipitated with TRPA1 (FLAG). HA‐tagged AQP4 and FLAG‐tagged TRPA1 were expressed in HEK293T cells. (j) Left, an illustration depicting the principle of the PLA. If the proteins of interest are in close proximity, the DNA probes hybridize to make circular DNA. DNA can be amplified and visualized by fluorescently labeled complementary oligonucleotide probes. Right, PLA result with anti‐AQP4 and anti‐TRPA1 antibodies. PLA signal was recognized as red fluorescence (λ_ex_ 594 nm, λ_em_ 624 nm), indicative of the proximity of AQP4 and TRPA1 (<40 nm). The red signal was pseudo‐colored as green for better data display. The lower panel is a negative control, with only anti‐TRPA1 antibody.

We next examined whether AQP4 silencing affects astrocytic Ca^2^^+^ signaling. Representative images and time‐course analyses showed that both brief and prolonged stimulation induced robust astrocytic Ca^2^^+^ responses in cortical slices, whereas these responses were attenuated following AQP4 silencing (Figure 2e–h). Quantitative analysis confirmed that AQP4 silencing significantly reduced both the peak amplitude and AUC of the astrocytic Ca^2^^+^ response following both stimulation durations (Figure 2g,h). Thus, AQP4 is required for the neuronal activity‐dependent astrocytic Ca^2^^+^ signaling in the S1FL.

Having found that astrocytic AQP4 gene silencing reduced both activity‐dependent volume changes and Ca^2+^ responses, we hypothesized that AQP4‐dependent changes in astrocyte volume or membrane tension might modulate TRPA1, a mechanosensitive Ca^2^^+^‐permeable channel. We therefore examined whether astrocytic AQP4 and TRPA1 are biochemically and spatially associated. Co‐immunoprecipitation (IP) assay in a heterologous expression system revealed that HA‐tagged AQP4 was immunoprecipitated with TRPA1 (FLAG) (Figure 2i), indicating an association between AQP4 and TRPA1 within the same protein complex. To determine whether these two proteins are also located in close proximity in the S1FL, we implemented a proximity ligation assay (PLA) to visualize protein‐protein interaction with high specificity and sensitivity in fixed brain slices. Consistent with the co‐IP results, we observed a positive PLA signal in the presence of both AQP4 and TRPA1 antibodies but not when the AQP4 antibody was omitted (Figure 2j). These findings support a biochemical and spatial association between AQP4 and TRPA1 proteins. Although they do not establish direct physical or functional interactions, these results raise the possibility that AQP4 and TRPA1 may participate in a shared signaling mechanism.

### 2.3 Astrocytic TRPA1 is Selectively Required for Ca^2+^ Signaling and Arteriolar Dilation During Prolonged Sensory Stimulation

To determine whether astrocytic TRPA1 contributes to sensory‐evoked astrocytic Ca^2^^+^ signaling and penetrating arteriole dilation, we simultaneously monitored astrocytic Ca^2^^+^ activity and penetrating arteriole diameter using in vivo two‐photon microscopy during brief (5 s) and prolonged (20 s) sensory stimulation. To induce astrocyte‐specific TRPA1 knockdown while monitoring astrocytic Ca^2^^+^ signals, we co‐injected AAVs expressing Cre‐dependent *Trpa1* shRNA, GFAP‐driven Cre, and gfaABC1D‐Lck‐GCaMP6f into the S1FL (Figure 3a). At least 2 weeks after viral injection, Texas Red‐dextran was retro‐orbitally administered to visualize the cerebral vasculature, allowing simultaneous imaging of membrane‐associated astrocytic Ca^2^^+^ signals and penetrating arteriole diameter (Figure 3b).

**FIGURE 3:**
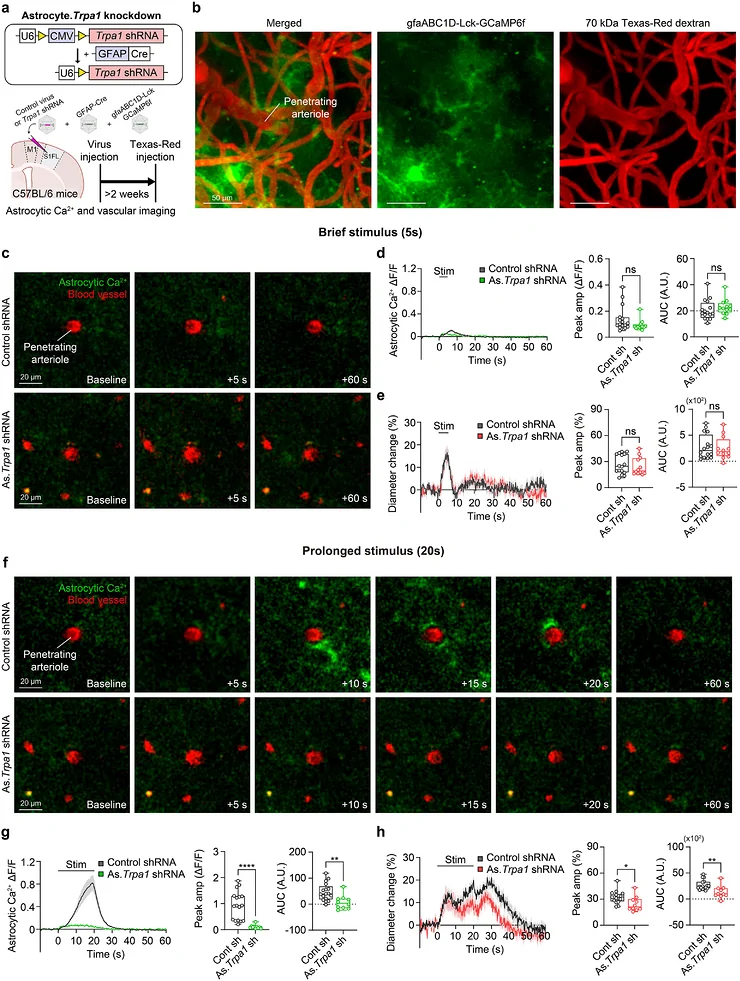
Effects of astrocytic TRPA1 knockdown on sensory‐evoked astrocytic Ca^2+^ activity and penetrating arteriole responses. (a) Schematic representation of the experimental design for simultaneous two‐photon imaging of astrocytic Ca^2^^+^ activity and vascular responses. For astrocyte‐specific TRPA1 knockdown, C57BL/6 mice received co‐injection of ‐GFAP‐Cre, gfaABC1D‐Lck‐GCaMP6f, and either a Cre‐dependent scrambled shRNA virus as the control or a Cre‐dependent *Trpa1* shRNA virus into the S1FL cortex. More than 2 weeks after viral injection, the cerebral vasculature was labeled by intravenous injection of 70‐kDa Texas Red–dextran. (b) Representative two‐photon images showing astrocytic GCaMP6f fluorescence, Texas Red–labeled cerebral vessels, and the merged image. The arrow indicates a penetrating arteriole. (c) Representative time‐lapse images of astrocytic Ca^2^^+^ activity and penetrating arteriole diameter during brief forepaw stimulation in control shRNA and astrocyte‐specific TRPA1 knockdown (As.*Trpa1* shRNA) mice. (d) Average time‐course traces of sensory‐evoked astrocytic Ca^2^^+^ responses during brief stimulation and quantification of the peak amplitude and AUC (Control shRNA; *n*  = 16, As.*Trpa1* shRNA; *n*  = 12, Peak amplitude; *p* = 0.119, AUC; *p* = 0.3862, ns, not significant, Unpaired t‐test). (e) Average time‐course traces of penetrating arteriole diameter changes during brief stimulation and quantification of the peak amplitude and AUC (Control shRNA; *n*  = 16, As.*Trpa1* shRNA; *n*  = 12, Peak amplitude; *p* = 0.5806, AUC; *p* = 0.7578, ns, not significant, Unpaired t‐test). (f) Representative time‐lapse images of astrocytic Ca^2^^+^ activity and penetrating arteriole diameter during prolonged forepaw stimulation in control shRNA and As.*Trpa1* shRNA mice. (g) Average time‐course traces and quantitative analyses of sensory‐evoked astrocytic Ca^2^^+^ responses during prolonged stimulation (Control shRNA; *n*  = 17, As.*Trpa1* shRNA; *n*  = 10, Peak amplitude; ^****^p < 0.0001, AUC; ^**^ *p* = 0.0038, Unpaired t‐test). (h) Average time‐course traces and quantitative analyses of penetrating arteriole diameter changes during prolonged stimulation (Control shRNA; *n*  = 17, As.*Trpa1* shRNA; *n*  = 10, Peak amplitude; ^*^ *p* = 0.0160, AUC; ^**^ *p* = 0.0033, Unpaired t‐test).

During brief sensory stimulation, modest astrocytic Ca^2^^+^ responses were observed in both control and astrocyte‐specific TRPA1 knockdown groups (Figure 3c,d). TRPA1 knockdown did not significantly alter either the peak amplitude or area under the curve (AUC) of the astrocytic Ca^2^^+^ response (Figure 3d). The brief stimulation elicited vasodilation of penetrating arterioles in both control and TRPA1 knockdown groups (Figure 3e). Importantly, the peak dilation and the AUC of the penetrating arteriole response were comparable between the groups during brief stimulation (Figure 3c,e), suggesting that astrocytic TRPA1 is less involved in regulating astrocytic Ca^2^^+^ and penetrating arteriole responses to brief sensory stimulation.

In contrast, prolonged sensory stimulation elicited a robust and sustained astrocytic Ca^2^^+^ response in control mice, whereas this response was markedly attenuated following astrocyte‐specific TRPA1 knockdown (Figure 3f,g). Quantitative analysis revealed significant reductions in both the peak amplitude and AUC of the astrocytic Ca^2^^+^ response in the TRPA1 knockdown group (Figure 3g). In addition, astrocyte‐specific TRPA1 knockdown significantly reduced the peak dilation and AUC of the penetrating arteriole responses during prolonged stimulation (Figure 3f,h). Together, these findings demonstrate that the TRPA1‐dependent component of astrocytic Ca^2^^+^ signaling is preferentially engaged during prolonged sensory stimulation and that astrocytic TRPA1 contributes to the accompanying dilation of penetrating arterioles.

### 2.4 Astrocytic TRPA1 is Necessary for CBV, Neuronal Activity, and Sensory Perception Following Prolonged Stimulation

We hypothesized that astrocytic TRPA1 plays a crucial role in maintaining CBV responses, neural activity, and sensory perception following AQP4‐mediated astrocytic volume changes during prolonged stimulation. In this experiment, we utilized the pSico virus system [45] to selectively silence the astrocytic TRPA1 gene [29] in non‐transgenic mice (C57BL/6 mice). At least 2 weeks following viral injection (a mixture of AAV‐GFAP‐mCherry‐Cre and AAV‐pSico‐*Trpa1* shRNA‐GFP), we performed IOS imaging as shown in AQP4 experiments (Figure 4a). Following brief stimulation, astrocytic TRPA1 knockdown did not alter CBV (Figure 4b, Figure S5c). However, peak amplitude, time to half maximum, and AUC of CBV were significantly diminished following prolonged stimulation in astrocytic TRPA1 knockdown mice compared to control mice (Figure 4c, Figure S5c). These results indicate that astrocytic TRPA1 is necessary for the magnitude and sustainability of CBV following prolonged sensory stimulation, but not in brief sensory stimulation. To determine whether the altered CBV response could be attributed to differences in anesthesia depth, we additionally examined sensory‐evoked responses under graded isoflurane anesthesia (Figure S8). Because 0.5% isoflurane was too light for forepaw stimulation, whisker air‐puff stimulation was used instead (Figure S8a). Anesthesia depth primarily affected CBV amplitude while preserving the overall temporal profile (Figure S8b,c), whereas astrocytic TRPA1 knockdown induced selective attenuation of the late‐phase response. These findings indicate that the altered CBV kinetics were not simply attributable to differences in anesthesia depth but instead reflect an astrocyte TRPA1‐dependent modulation of the late‐phase response that shapes the temporal dynamics of sensory‐evoked CBV.

**FIGURE 4:**
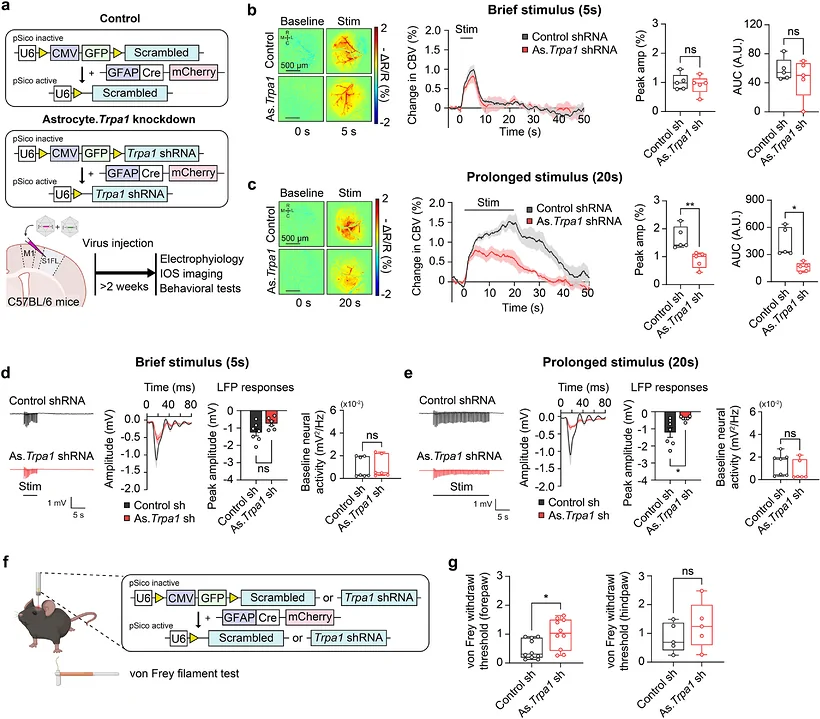
Effects of astrocytic TRPA1 knockdown on sensory‐evoked neural activities, CBV, and sensory perception. (a) Schematic illustrations of the injection of pSico AAV virus carrying either control shRNA or *Trpa1* shRNA along with GFAP‐mCherry‐Cre virus into the mouse S1FL cortex for genetic ablation of the astrocyte‐specific TRPA1 channel. (b) Representative spatiotemporal IOS images, the trace of CBV changes, peak amplitude (%), and area under the curve during brief stimulation in control and astrocytic TRPA1 knockdown mice (Control shRNA; *n*  = 5, As.*Trpa1* shRNA; *n*  = 5, Peak amplitude; *p* = 0.64, AUC; *p* = 0.41, ns, not significant, Unpaired t‐test). (c) Representative spatiotemporal IOS images, the trace of CBV changes, peak amplitude (%), and area under the curve during prolonged stimulation in control and astrocytic TRPA1 knockdown mice (Control shRNA; *n*  = 5, As.*Trpa1* shRNA; *n*  = 5, Peak amplitude; ^**^ *p* = 0.0080, AUC; ^*^ *p* = 0.015, Unpaired t‐test). (d) Representative LFP traces, amplitudes, and peak amplitude during brief stimulation and basal neuronal activity before brief stimulation (Control shRNA; *n*  = 7, As.*Trpa1* shRNA; *n*  = 6, Peak amplitude; *p* = 0.065, Baseline activity; *p* = 0.94, ns, not significant, Unpaired t‐test). (e) Representative LFP traces, amplitudes, and peak amplitude during prolonged stimulation and basal neuronal activity before brief stimulation (Control shRNA; *n*  = 7, As.*Trpa1* shRNA; *n*  = 6, Peak amplitude; ^*^ *p* = 0.025, Baseline activity; *p* = 0.30, ns, not significant, Unpaired t‐test). (f) Illustration of the von Frey filament test. (g) Average von Frey withdrawal threshold in forepaw stimulation and hindpaw stimulation (Control shRNA; *n*  = 5, As.*Trpa1* shRNA; *n*  = 5, Forepaw; ^*^ *p* = 0.015, Hindpaw; *p* = 0.35, ns, not significant, Unpaired t‐test).

To compare the temporal pattern of the CBV response in global knockout (KO) mice with that observed following astrocyte‐specific TRPA1 knockdown, we measured CBV responses in global TRPA1 KO mice. We observed that whereas astrocyte‐specific TRPA1 knockdown selectively reduces the late phase of the CBV response during prolonged stimulation while preserving the early response, global TRPA1 KO mice exhibited earlier and broader changes during both brief and prolonged stimulation (Figure S9). This distinct temporal pattern may arise from non‐astrocytic effects, as well as developmental or compensatory changes, indicating that the selective late‐phase reduction is specific to astrocytic TRPA1, whereas global deletion likely reflects additional contributions from other cell types. These results further highlight that the selective late‐phase reduction following astrocyte‐specific knockdown reflects an astrocyte‐mediated component of the CBV response.

We then assessed whether astrocytic TRPA1 impacts neuronal activity during sensory stimulation by recording LFP. The basal neuronal activity before sensory stimulation showed no significant difference with astrocytic TRPA1 knockdown (Figure 4d,e). During brief stimulation, neuronal activity was not changed in astrocytic TRPA1 knockdown mice compared to control mice (Figure 4d). On the other hand, neuronal activity was significantly disturbed following prolonged stimulation in astrocytic TRPA1 knockdown mice compared to control mice (Figure 4e). These results demonstrate that the astrocytic TRPA1 channel controls neuronal activity during prolonged sensory stimulation, similar to the AQP4 channel.

Next, we examined whether astrocytic TRPA1 knockdown in S1FL impairs sensory perception using the von Frey test (Figure 4f). We confirmed that the hind‐paw stimulation did not affect the withdrawal threshold (Figure 4g) and that basal locomotion activity remained unchanged in these mice (Figure S6a,c). In contrast, the von Frey withdrawal threshold was significantly increased following forepaw stimulation in the astrocytic TRPA1 knockdown mice (Figure 4g). This result indicates that astrocytic TRPA1 contributes to sensory perception, similar to AQP4. Taken together, astrocytic TRPA1 controls CBV, neuronal activity, and sensory perception following prolonged sensory stimulation, supporting a functional role for astrocytic TRPA1 downstream of astrocytic volume changes.

### 2.5 Astrocytic TRPA1 is Necessary for D‐Serine Release

Astrocytic TRPA1 regulates not only basal Ca^2+^ levels but also the D‐serine tone that is required for NMDAR activation in the brain [46]. Given these insights, we hypothesized that astrocytic TRPA1 modulates sensory‐evoked neuronal activity through D‐serine release, which, in turn, influences CBV. To study the effects of D‐serine on neuronal activity, we measured 2‐Amino‐5‐phosphono‐pentanoic acid (APV, a GluN2 subunit‐specific NMDAR antagonist)‐sensitive tonic NMDAR currents in S1FL of astrocytic TRPA1 knockdown mice (Figure 5a). We found that tonic NMDAR currents were significantly reduced in the astrocytic TRPA1 knockdown mice (Figure 5b,c), indicating that D‐serine release requires TRPA1. Next, we bath‐applied D‐serine to saturate the NMDAR glycine modulatory site (GMS). We found that the baseline current shift was higher in the astrocytic TRPA1 knockdown mouse after the D‐serine treatment (ΔD‐serine) (Figure 5b,d). In line with this result, tonic NMDAR GMS occupancy was significantly lower in the astrocytic TRPA1 knockdown mice compared to the control mice (Figure 5e). To clarify the cellular source of D‐serine, we selectively silenced serine racemase (SR), an enzyme converting L‐serine to D‐serine, in astrocytes or neurons using cell type‐specific Cre drivers (GFAP‐Cre and CaMKIIα‐Cre). Tonic NMDAR currents were not significantly affected in either group (Figure 5f,g). However, we observed an elevation of ΔD‐serine currents in the astrocytic SR knockdown mice when compared to the control mice (Figure 5h). Likewise, tonic NMDAR GMS occupancy was significantly reduced in the astrocytic SR knockdown mice (Figure 5i), indicating a reduction in basal D‐serine levels in these mice. On the other hand, neuronal SR knockdown did not significantly alter ΔD‐serine currents or tonic NMDAR GMS occupancy (Figure 5h,i), suggesting that neuronal SR makes a limited contribution to the maintenance of basal D‐serine levels. Collectively, these results demonstrate that astrocytic TRPA1 is a key contributor to neuronal tonic NMDAR currents through D‐serine release.

**FIGURE 5:**
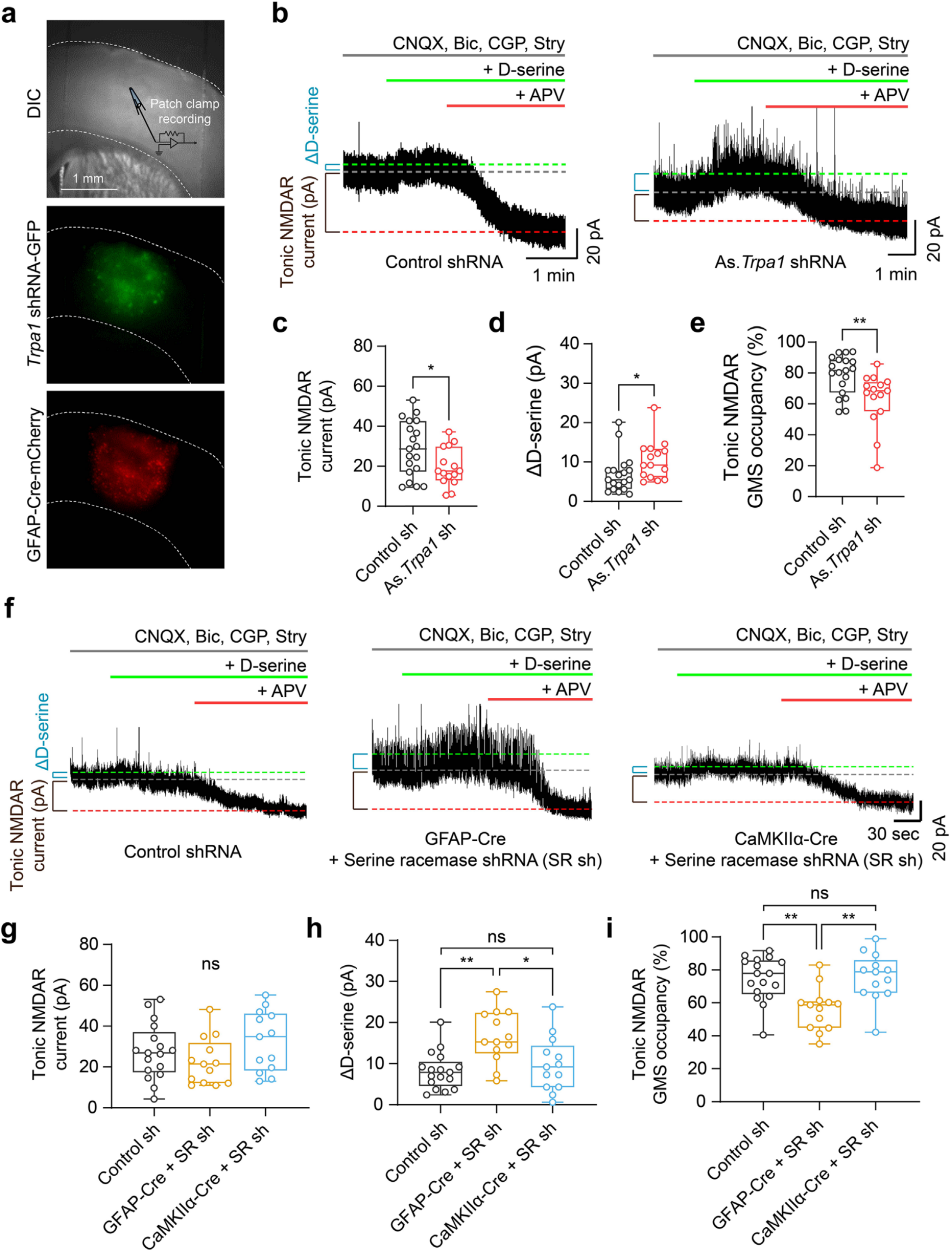
Regulation of astrocytic TRPA1 on D‐serine and NMDAR tone in the S1FL cortex. (a) Representative images of DIC, GFP (pSico‐*Trpa1* shRNA), and RFP (GFAP‐mCherry‐Cre). (b) Representative traces of tonic NMDAR currents in control and TRPA1 knockdown mice. (c–e) Summary bar graph of tonic NMDAR current, ΔD‐serine, and tonic NMDAR GMS occupancy in control and TRPA1 knockdown mice (Control shRNA; *n*  = 19, As.*Trpa1* shRNA; *n*  = 15, Tonic NMDAR current; ^*^ *p* = 0.034, ΔD‐serine; ^*^ *p* = 0.044, Tonic NMDAR GMS occupancy; ^**^ *p* = 0.0057, ns, not significant, Unpaired t‐test). (f) Representative trace of tonic NMDAR current in control shRNA, astrocytic serine racemase knockdown, and neuronal serine racemase knockdown mice. (g–i) Summary bar graph of tonic NMDAR current, ΔD‐serine, and tonic NMDAR GMS occupancy in control shRNA, astrocytic serine racemase knockdown, and neuronal serine racemase knockdown mice (Control shRNA; *n*  = 17, GFAP‐Cre + SR shRNA; *n*  = 13, CaMKIIα‐Cre + SR shRNA; *n*  = 13, Tonic NMDAR current; Control shRNA vs. GFAP‐Cre + SR shRNA; *p*  = 0.57, Control shRNA vs. CaMKIIα‐Cre + SR shRNA; *p*  = 0.56, GFAP‐Cre + SR shRNA vs. CaMKIIα‐Cre + SR shRNA; *p*  = 0.15, ΔD‐serine; Control shRNA vs. GFAP‐Cre + SR shRNA; ^**^ *p*  = 0.0010, Control shRNA vs. CaMKIIα‐Cre + SR shRNA; *p*  = 0.75, GFAP‐Cre + SR shRNA vs. CaMKIIα‐Cre + SR shRNA; ^*^ *p*  = 0.013, Tonic NMDAR GMS occupancy; Control shRNA vs. GFAP‐Cre + SR shRNA; ^**^ *p*  = 0.0019, Control shRNA vs. CaMKIIα‐Cre + SR shRNA; *p*  = 0.98, GFAP‐Cre + SR shRNA vs. CaMKIIα‐Cre + SR shRNA; ^**^ *p*  = 0.0021, ns, not significant, one‐way ANOVA, Tukey's multiple comparisons test).

### 2.6 D‐Serine Restores Impaired CBV, Neuronal Activity, and Sensory Perception in Astrocytic TRPA1 Knockdown Mice

Based on the findings of the regulatory role of astrocytic TRPA1 through D‐serine, we investigated whether D‐serine could restore the impaired CBV, neuronal activity, and sensory perception observed during the prolonged stimulation in astrocytic TRPA1 knockdown mice. D‐serine has been reported to cross the blood‐brain barrier (BBB) [47]. Therefore, we administered D‐serine intraperitoneally (600 mg/kg) to mice with astrocytic TRPA1 knockdown (Figure 6a). D‐serine was delivered 2 h before the in vivo experiments to eliminate direct effects of systemic D‐serine on vascular tone. This dose is reported to be effective in mice and is approximately equivalent to human doses (>30 mg/kg) [48, 49]. Given that astrocytic D‐serine directly induces dilation of parenchymal arterioles [50], we first tested D‐serine effects on CBV in control shRNA mice. D‐serine did not affect CBV in control mice during both brief and prolonged forepaw stimulation (Figure 6a–c), indicating that D‐serine does not exert a direct vasodilatroy effect after 2 h of administration. Then, we next administered D‐serine to astrocytic TRPA1 knockdown mice. In brief stimulation, we observed no significant change in CBV between the vehicle and D‐serine‐treated groups (Figure 6d). In contrast, we observed a significant increase in CBV following prolonged stimulation in the D‐serine‐treated astrocytic TRPA1 knockdown group, showing full restoration of the peak amplitude, time to half maximum, and AUC of CBV to the level observed in control shRNA mice (Figure 6e, Figure S5d). These rescue effects provide functional evidence that D‐serine remained effective at the time of recording, although we have not directly measured the local D‐serine concentration in S1FL. Furthermore, the results demonstrate that D‐serine is sufficient to restore impaired CBV responses in astrocytic TRPA1 knockdown mice during prolonged stimulation, supporting a role for astrocytic TRPA1 in maintaining CBV responses during prolonged stimulation through D‐serine release.

**FIGURE 6:**
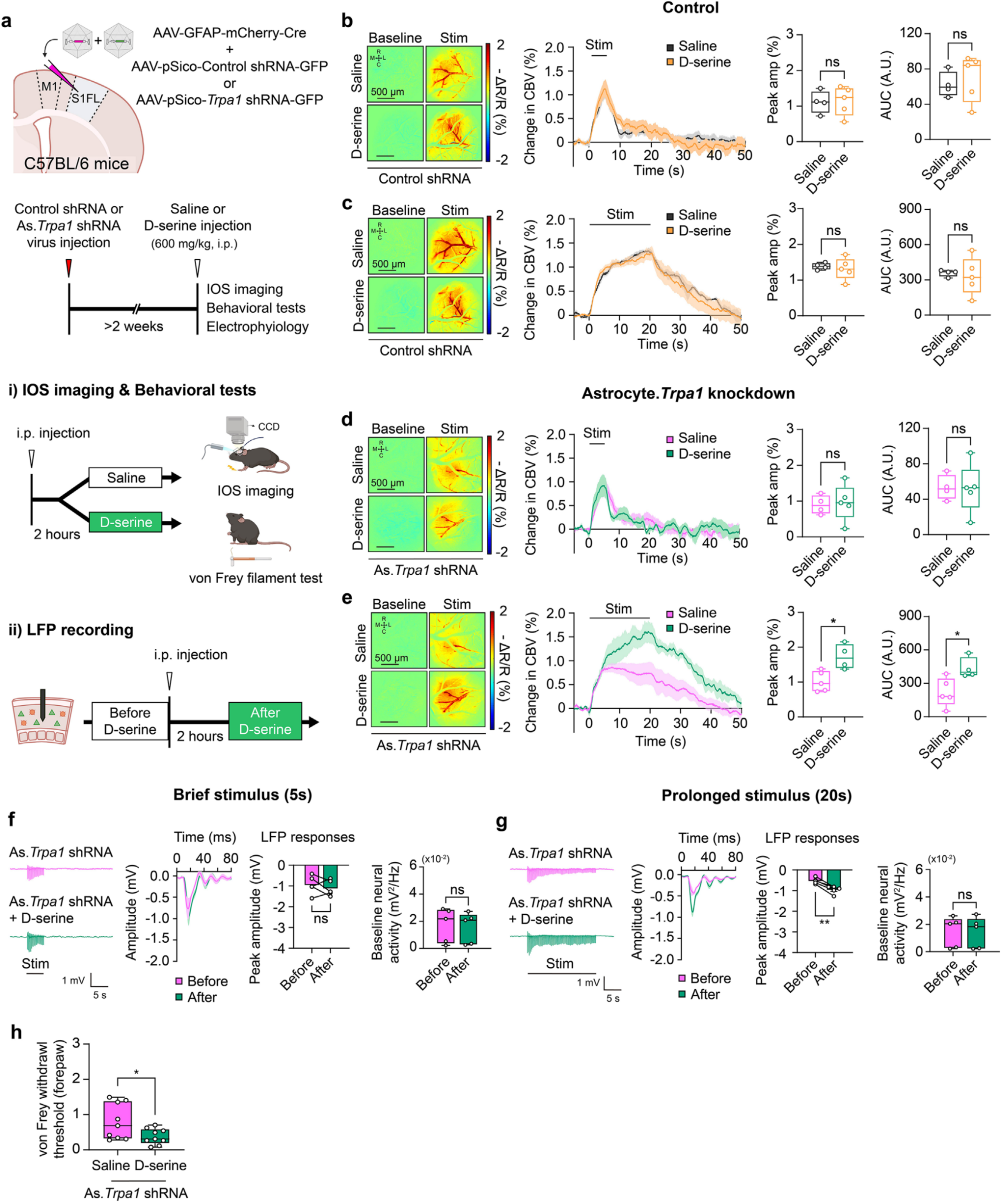
Rescue effect of D‐serine treatment on sensory‐evoked neural activities, CBV, and sensory perception threshold in astrocyte‐specific TRPA1 knockdown mice. (a) Schematic illustrations of injecting pSicoR AAV virus carrying control shRNA or *Trpa1* shRNA into the mouse S1FL and a timeline of the experiments. i, ii). Experimental procedures for intrinsic optical signal (IOS) imaging, von Frey filament test, and local field potential (LFP) recording. (b) Representative spatiotemporal IOS images, the trace of CBV changes, peak amplitude (%), and area under the curve before and after D‐serine treatment during brief stimulation in astrocytic TRPA1 scrambled mice (Control shRNA + Saline; *n*  = 4, Control shRNA + D‐serine; *n*  = 5, Peak amplitude; *p* = 0.90, AUC; *p* = 0.62, ns, not significant, Unpaired t‐test). (c) Representative spatiotemporal IOS images, the trace of CBV changes, peak amplitude (%), and area under the curve before and after D‐serine treatment during prolonged stimulation in astrocytic TRPA1 scrambled mice (Control shRNA + Saline; *n*  = 4, Control shRNA + D‐serine; *n*  = 5, Peak amplitude; *p* = 0.68, AUC; *p* = 0.76, ns, not significant, Unpaired t‐test). (d) Representative spatiotemporal IOS images, the trace of CBV changes, peak amplitude (%), and area under the curve before and after D‐serine treatment during brief stimulation in astrocytic TRPA1 knockdown mice (As.*Trpa1* shRNA + Saline; *n*  = 4, As.*Trpa1* shRNA + D‐serine; *n*  = 5, Peak amplitude; *p* = 0.83, AUC; *p* = 0.94, ns, not significant, Unpaired t‐test). (e) Representative spatiotemporal IOS images, the trace of CBV changes, peak amplitude (%), and area under the curve before and after D‐serine treatment during prolonged stimulation in astrocytic TRPA1 knockdown mice (As.*Trpa1* shRNA + Saline; *n*  = 5, As.*Trpa1* shRNA + D‐serine; *n*  = 4, Peak amplitude; ^*^ *p* = 0.014, AUC; ^*^ *p* = 0.026, Unpaired t‐test). (f) Representative LFP traces, amplitudes, and peak amplitude before and after D‐serine treatment during brief stimulation and basal neuronal activity (*n*  = 5, Peak amplitude; *p* = 0.48, Baseline activity; *p* = 0.21, ns, not significant, Paired t‐test). (g) Representative LFP traces, amplitudes, and peak amplitude before and after D‐serine treatment during prolonged stimulation and basal neuronal activity (*n*  = 5, Peak amplitude; ^**^ *p* = 0.0013, Baseline activity; *p* = 0.83, ns, not significant, Paired t‐test). (h) Average von Frey withdrawal threshold in forepaw stimulation for astrocytic TRPA1 knockdown mice treated with saline or with D‐serine (As.*Trpa1* shRNA + Saline; *n*  = 9, As.*Trpa1* shRNA + D‐serine; *n*  = 9, ^*^ *p* = 0.044, Unpaired t‐test).

Next, we tested whether D‐serine restores impaired neuronal activity observed in astrocytic TRPA1 knockdown mice to prolonged stimulation (Figure 6a). D‐serine administration showed no significant difference in basal neuronal activity before both brief and prolonged stimulation (Figure 6f,g). While D‐serine treatment did not affect neuronal activity following brief stimulation in astrocytic TRPA1 knockdown mice (Figure 6f), we observed that D‐serine fully restored the reduced peak amplitude of neuronal activity following prolonged stimulation compared to the saline‐treated astrocytic TRPA1 knockdown group (Figure 6g). These results indicate that D‐serine is sufficient to restore impaired neuronal activity in astrocytic TRPA1 knockdown mice to the control level observed in control scrambled shRNA mice (Figure 4e), suggesting that astrocytic TRPA1 plays a crucial role in enhancing and maintaining neuronal activity following prolonged sensory stimulation through D‐serine.

We next investigated whether D‐serine can rescue impaired sensory perception in astrocytic TRPA1 knockdown mice. 2 h before performing the von Frey test, D‐serine or vehicle was injected intraperitoneally (Figure 6a). We found that D‐serine administration fully rescued the withdrawal threshold following forepaw stimulation in astrocytic TRPA1 knockdown mice (Figure 6h) to the control level observed in control scrambled shRNA mice (Figure 4g). This result demonstrates that astrocytic TRPA1 significantly contributes to sensory perception following prolonged stimulation through D‐serine. Taken together, astrocytic TRPA1 boosts CBV responses, neuronal activity, and sensory perception following prolonged sensory stimulation through D‐serine release.

### 2.7 Astrocytic D‐Serine Directly Controls Both Neuronal NMDAR Currents and Basal Tones of Penetrating Arterioles via BEST1

Based on the findings that astrocytic D‐serine regulates neuronal tonic NMDAR tones, we investigated whether a direct infusion of D‐serine via a patch pipette affects APV‐sensitive neuronal tonic NMDAR currents in acute brain slices. To infuse D‐serine (0 or 20 mm) into a gap‐junction‐coupled astrocytic syncytium, we performed a whole‐cell patch‐clamp recording in a single astrocyte, allowed D‐serine to diffuse for at least 30 min, and measured tonic NMDAR currents in neurons near the patched astrocyte (<200 µm) in S1FL (Figure 7a,b). 20 mm D‐serine infusion in astrocytes significantly increased neuronal tonic NMDAR currents compared to 0 mM D‐serine infusion in C57BL/6 mice (Figure 7c,d), indicating that astrocytic D‐serine directly mediates neuronal tonic NMDAR currents in S1FL. Given that TRPA1‐mediated Ca^2+^ increases in astrocytes can activate the BEST1 channel [10], and that CBV responses were disrupted during prolonged stimulation in BEST1 KO mice (Figure S10), we further investigated whether astrocytic D‐serine is released through the BEST1 channel to regulate neuronal tonic NMDAR currents. We measured neuronal tonic NMDAR currents in wild‐type (WT) and BEST1 knockout (KO) mice following D‐serine infusion in astrocytes and found that D‐serine infusion in astrocytes did not increase tonic NMDAR currents in BEST1 KO mice compared to WT mice (Figure 7e,f). Similarly, in astrocyte‐selective BEST1 knockout mice [51], D‐serine infusion failed to enhance neuronal NMDAR currents compared to WT mice (Figure 7g,h). The 20 mm D‐serine‐mediated tonic NMDAR currents in BEST1 KO mice were similar to the 0 mm D‐serine‐mediated tonic NMDAR currents observed in control mice. Taken together, these results demonstrate that astrocytic D‐serine release through the BEST1 channel controls neuronal tonic NMDAR currents.

**FIGURE 7:**
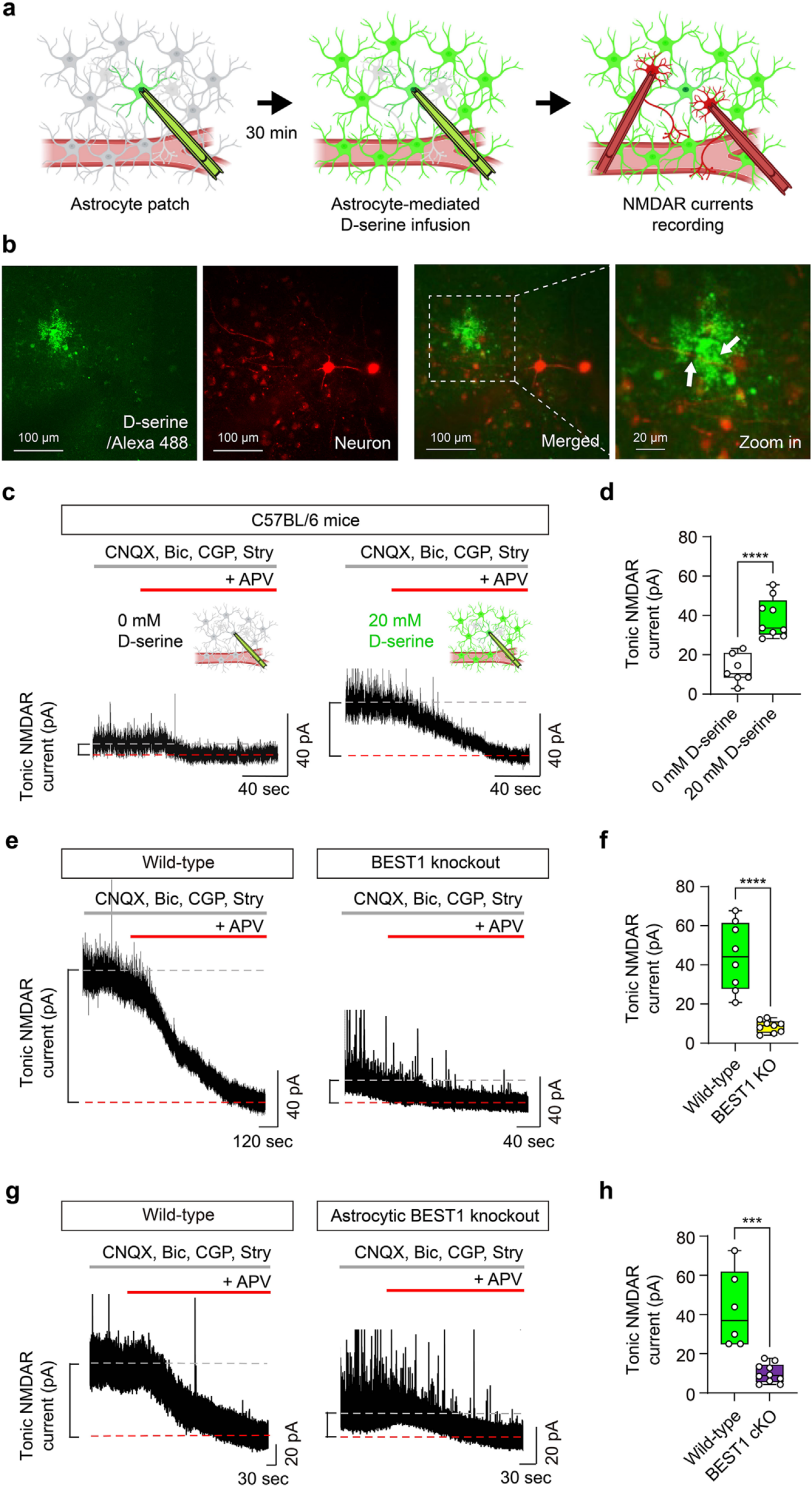
Regulatory role of astrocytic BEST1/D‐serine signaling on neuronal NMDA tones in S1FL. (a) Experimental illustration of astrocytic Alexa 488/D‐serine (green) loading through astrocytic gap‐junction‐coupled syncytium and neuronal patch (red). (b) Representative confocal images of astrocytes with Alexa 488 and neurons with Alexa 555. Note that astrocytic processes are in close proximity to neuronal dendrites (arrowheads). (c, d) Representative traces and summary bar graph of tonic NMDAR current after 0‐ or 20‐mM D‐serine infusion in astrocytes of C57BL/6 mice (0 mm; *n*  = 7, 20 mm; *n*  = 9, ^****^ *p* < 0.0001, Unpaired t‐test). (e, f) Representative traces and summary bar graph of tonic NMDAR current after 20‐mM D‐serine infusion in astrocytes of BEST1 KO mice (WT; *n*  = 8, BEST1 KO; *n*  = 9, ^****^ *p* < 0.0001, Unpaired t‐test). (g, h) Representative traces and summary bar graph of tonic NMDAR current after 20‐mM D‐serine infusion in astrocytes of astrocytic BEST1 knockdown mice (WT; *n*  = 8, BEST1 cKO; *n*  = 9, ^***^ *p* = 0.0002, Unpaired t‐test).

Among the NVU, astrocytic endfeet enwrap penetrating arterioles and regulate the vascular tone. Based on this, we hypothesized that astrocytic D‐serine controls the basal vascular tone of penetrating arterioles through the BEST1 channel. To test this hypothesis, we measured diameter changes of penetrating arterioles before and after D‐serine (20 mm) infusion into astrocytes in brain slices (Figure 8a). D‐serine was loaded into astrocytes through the patch pipette during whole‐cell patch‐clamp recordings. We utilized ex vivo brain slices, lacking neuronal activity, to assess basal vascular tone, which we propose to be regulated by astrocytic D‐serine. To induce comparable vascular tone of penetrating arterioles in WT and BEST1 KO mice, we cannulated penetrating arterioles and then bath‐applied thromboxane A2R agonist (TXA2), U46619 (100 nm) [52, 53]. We observed that the cannulated arteriole in WT mice progressively dilated upon astrocytic D‐serine infusion via whole‐cell configuration, but not in BEST1 KO mice (Figure 8b–e), directly indicating that BEST1 is required for astrocytic D‐serine release and vasodilation. Also, we observed that kynurenic acid (KA, a GluN1 subunit‐specific NMDAR antagonist, 1 mm) treatment constricted the cannulated arterioles below the basal tone in WT mice, but not in BEST1 KO mice, indicating that the GluN1 subunit‐containing NMDAR is necessary for the maintenance of vascular tone in penetrating arterioles in S1FL (Figure 8b–d). A significant difference in total vasoreactivity (dilation + constriction) between the two groups was also observed (Figure 8e). These results indicate that astrocytic D‐serine controls and maintains basal vascular tone of penetrating arterioles through both the BEST1 and GluN1 subunit‐containing NMDAR. It is possible that D‐serine may bind neuronal NMDAR, as well as endothelial NMDAR. To test this possibility and clarify cell type‐specific responses to D‐serine, we measured vessel diameter changes in cannulated arterioles before and after D‐serine injection into astrocytes in the presence of TTX to eliminate neuronal contribution. D‐serine diffusion through astrocytic gap junction networks increased vessel diameter even when brain slices were incubated with TTX, and this effect was reduced by treatment with kynurenic acid, a competitive antagonist at the glycine‐binding site of NMDARs (Figure S11). These results indicate that D‐serine released from astrocytes can activate non‐neuronal NMDARs to induce vasodilation. Together with prior evidence demonstrating endothelial NMDA receptor expression [54, 55] and endothelial GluN1‐dependent vasodilation [37, 56], our results support the role of astrocyte‐derived D‐serine in facilitating endothelial NMDA receptor activation and downstream eNOS‐dependent vasodilation.

**FIGURE 8:**
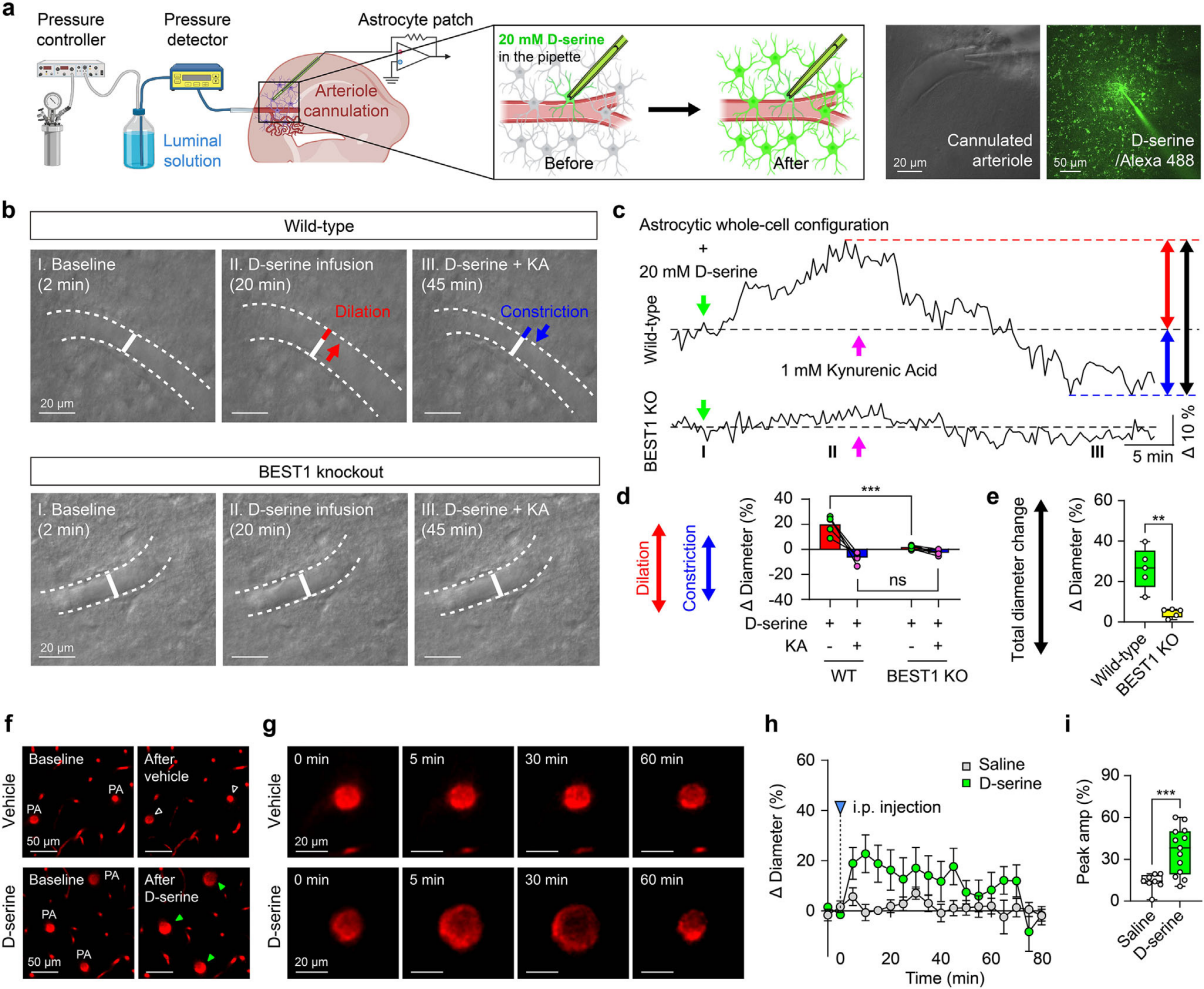
Regulatory role of astrocytic BEST1/D‐serine signaling on basal vascular tone. (a) Experimental illustration of penetrating arteriole cannulation and astrocytic Alexa 488/D‐serine (green) loading through astrocytic gap‐junction‐coupled syncytium in the brain slice, and DIC image of the cannulated arteriole and 2‐photon image of astrocytic D‐serine infusion. (b) Representative DIC images of changes in cannulated arteriole diameter before and after astrocytic D‐serine infusion and KA treatment in WT and BEST1 KO mice. (c) Representative traces of changes in cannulated arteriole diameter before and after astrocytic D‐serine infusion and KA treatment in WT and BEST1 KO mice. (d) Summary bar graph of Δ diameter by astrocytic D‐serine infusion and KA treatment, respectively in WT and BEST1 KO mice (WT; *n*  = 5, BEST1 KO; *n*  = 5, Unpaired t‐test). (e) Summary bar graph of total diameter (dilation + constriction) by astrocytic D‐serine infusion and KA treatment in WT and BEST1 KO mice (*n*  = 5, ^**^ *p* = 0.0015, Unpaired t‐test). (f) Representative in vivo 2‐photon images of penetrating arterioles in mice administered saline or D‐serine intraperitoneally. (g) Representative in vivo two‐photon images showing changes in penetrating arteriole diameter following intraperitoneal administration of saline or D‐serine. (h) Average data of diameter changes by saline and D‐serine IP injection (Saline; *n*  = 8, D‐serine; *n*  = 13, ^***^ *p* = 0.00043, Unpaired t‐test). (i) Average data of peak diameter changes by saline and D‐serine IP injection.

Finally, to further investigate whether D‐serine directly dilates penetrating arterioles in vivo, we performed in vivo two‐photon microscopic imaging to assess diameter changes of penetrating arterioles before and after administration of D‐serine or saline. We observed that D‐serine administration induced dilation of penetrating arterioles, whereas saline had no effect on their diameter (Figure 8f–i). These findings indicate that D‐serine directly modulates the basal vascular tone of penetrating arterioles in vivo, similar to ex vivo. Taken together, our results suggest that astrocytic D‐serine regulates basal vascular tone and CBV responses following prolonged sensory stimulation through the BEST1 channel.

## 3 Discussion

Our study provides a comprehensive mechanistic framework for understanding how astrocytes coordinate neuronal and vascular function during prolonged sensory demand and regulate basal vascular tone. Using astrocyte‐selective genetic manipulations targeting AQP4, TRPA1, serine racemase, and BEST1, we delineated the integrated molecular events that regulate CBV across different stimulation durations and how astrocytic volume change‐induced Ca^2+^ enhancement is involved (Figure 9a–c). Brief sensory stimulation induces transient neuronal activation and a transient CBV response (Figure 9a). In contrast, prolonged sensory stimulation evokes prolonged neuronal activation, followed by a profound astrocytic Ca^2+^ elevation through TRPA1, which strengthens and sustains neuronal and CBV responses (Figure 9b). This facilitatory effect was absent under astrocytic TRPA1 gene‐silencing and fully rescued after D‐serine application (Figure 9b), indicating that astrocytes are essential to maintain neuronal and CBV responses during prolonged sensory stimulation via integrated activation of astrocytic AQP4/TRPA1/BEST1/D‐serine signaling (Figure 9b,c). Furthermore, we have demonstrated that astrocytes can maintain and control the vascular tone of penetrating arterioles with tonic D‐serine release through BEST1, even in the absence of neuronal activity (Figure 9c). Together, these combined molecular and cellular findings support a new mechanistic model of neuro‐glia‐vascular regulation in sensory perception (Figure 9c).

**FIGURE 9:**
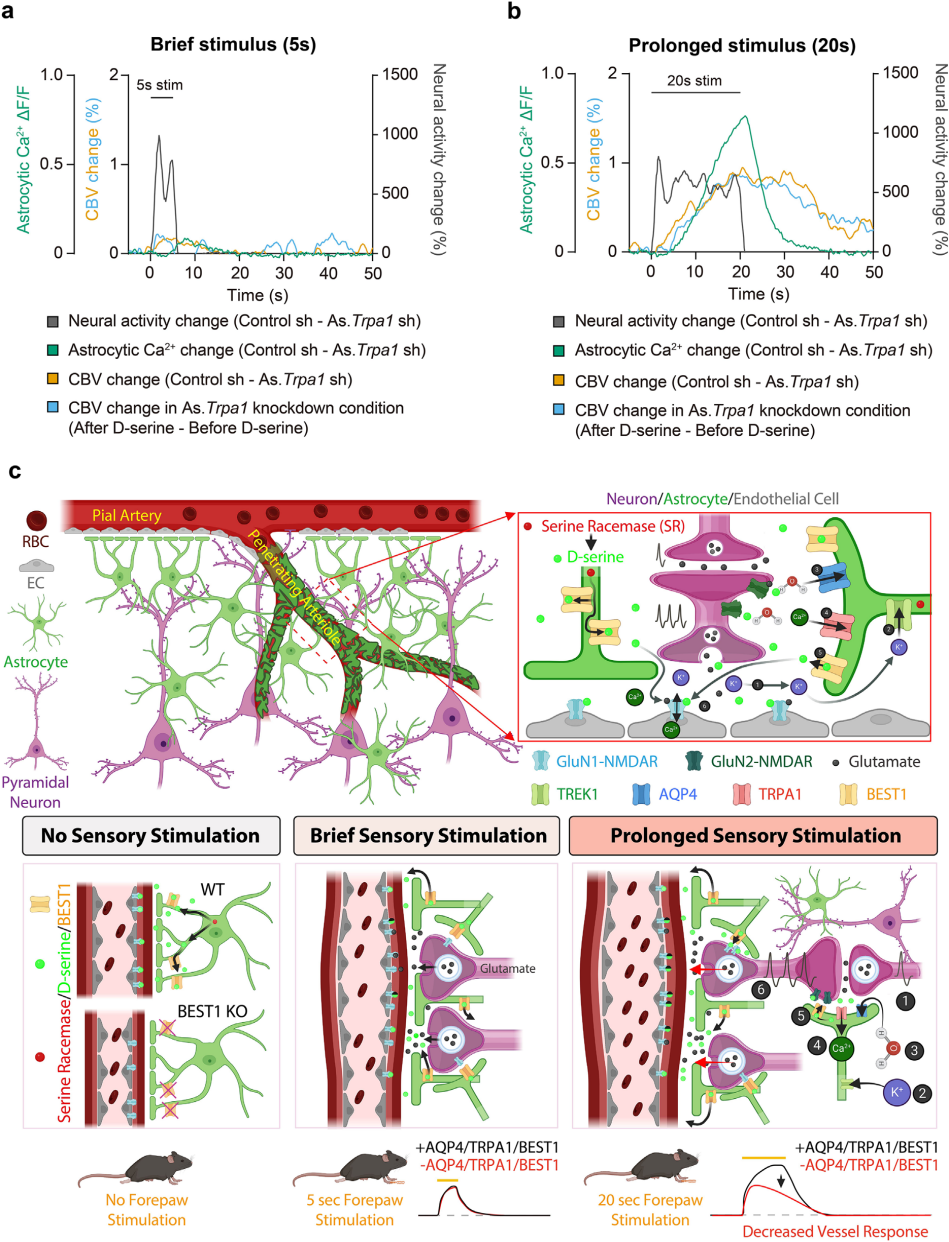
Proposed model of astrocytic AQP4/TRPA1/BEST1/D‐serine signaling during prolonged sensory stimulation in S1FL. (a) Subtraction traces of neuronal activity (gray) and CBV changes (orange: control mice‐astrocytic TRPA1 knockdown mice, blue: after‐before D‐serine IP injection in astrocytic TRPA1 knockdown mice) following brief stimulation. (b) Subtraction traces of neuronal activity (gray), astrocytic Ca^2+^ responses (green: control mice‐astrocytic TRPA1 knockdown mice), and CBV changes (orange: control‐astrocytic TRPA1 knockdown mice, blue: after‐before D‐serine IP injection in astrocytic TRPA1 knockdown mice) following prolonged stimulation. (c) Schematic diagram illustrating the integrated molecular mechanisms underlying astrocytic regulation of sensory‐evoked neural activity, CBV, and sensory perception via the AQP4/TRPA1/BEST1/D‐serine signaling pathway. The basal level of vascular tone (in the absence of sensory stimulation) is maintained by astrocytic SR/D‐serine/BEST1. Brief sensory stimulation induces vasodilation resulting from neuronal activation, not astrocytes. Prolonged sensory stimulation (1) induces the release of potassium ions (K^+^) from activated neurons. Astrocytes efficiently take up the released K^+^ ions (2), leading to an increase in cellular osmotic pressure. This osmotic pressure elevation promotes water influx through the aquaporin 4 (AQP4) channel (3) in astrocytes. Consequently, astrocytes undergo transient volume changes, activating the transient receptor potential ankyrin 1 (TRPA1) channel, leading to Ca^2+^ influx (4). Activation of TRPA1 causes the release of D‐serine (5) through BEST1 (6). Finally, the augmented extracellular levels of D‐serine boost neuronal activity by binding to GluN1‐containing NMDARs, leading to CBV changes.

The selective involvement of astrocyte AQP4/TRPA1/BEST1/D‐serine signaling during prolonged, but not brief, sensory stimulation raises the question of why this pathway exhibits such temporal selectivity. Our findings suggest that this selectivity may reflect differences in the magnitude and duration of AQP4‐mediated volume responses and their capacity to recruit TRPA1‐dependent Ca^2+^ signaling. Although both brief and prolonged stimulation induced AQP4‐dependent volume responses, prolonged stimulation produced larger and more sustained changes, which might more effectively recruit the downstream TRPA1‐dependent Ca^2^^+^ signaling (Figure 2). During sustained neuronal activity, the progressive accumulation of extracellular K^+^ and associated osmotic water influx may enhance astrocyte swelling and extracellular‐space shrinkage [57, 58], thereby promoting TRPA1‐dependent Ca^2^^+^ signaling through membrane‐, volume‐, or swelling‐associated mechanisms [9, 10, 59]. Consistent with this, prolonged stimulation evoked a robust and sustained astrocytic Ca^2^^+^ response that was reduced by TRPA1 gene silencing, whereas brief stimulation produced only a minor Ca^2^^+^ response (Figure 3). Thus, temporal selectivity may arise because the transient volume changes induced by brief stimulation are insufficient to engage TRPA1‐dependent signaling, whereas prolonged stimulation generates volume dynamics that more effectively recruit this pathway.

Although our data do not establish a discrete activation threshold or demonstrate direct gating of TRPA1 by astrocyte swelling, they support a model in which larger and more sustained AQP4‐dependent volume changes progressively recruit TRPA1‐dependent Ca^2^^+^ signaling. This Ca^2^^+^ elevation may promote BEST1‐mediated D‐serine release, thereby supporting NMDA receptor‐dependent neuronal excitation and the late phase of the CBV response. Accordingly, the AQP4/TRPA1/BEST1/D‐serine pathway may serve as a complementary mechanism, rather than initiating neurovascular responses, consistent with the selective reduction in the magnitude and persistence, but not the onset of prolonged responses following AQP4 or TRPA1 gene silencing. The remaining neuronal and CBV responses likely reflect parallel neuronal, vascular, and astrocytic pathways, potentially including GPCR‐ or IP_3_R‐dependent signaling and AQP4/TRPA1‐associated homeostatic mechanisms involving Kir4.1, GLT‐1, GAT‐3, and TRPV4 [26, 60, 61, 62, 63]. These mechanisms may work together to maintain ionic and neurotransmitter homeostasis, neuronal activity, and vascular responses during sustained stimulation [12, 64].

Our study proposes two distinct, context‐dependent roles for astrocyte‐derived D‐serine. During prolonged sensory stimulation, D‐serine release from astrocytes helps sustain neuronal excitability by enhancing neuronal NMDA receptor currents. Our previous study demonstrated that BEST1‐mediated gliotransmitter release from hippocampal astrocytes regulates basal NMDAR tone and synaptic plasticity [30]. In line with this, it has been reported that glial D‐serine regulates NMDAR co‐agonist‐site occupancy and synaptic plasticity [32]. Our data support neuronal NMDA receptors as the downstream target of astrocyte‐derived D‐serine during sensory stimulation. Both astrocyte‐specific TRPA1 and BEST1 knockdown reduced APV‐sensitive tonic NMDA receptor currents and glycine modulatory site occupancy in S1FL neurons (Figures 5 and 7). Consistently, in vivo LFP recordings showed that the reduction in neuronal activity during the prolonged stimulation (20 s) following astrocytic TRPA1 gene silencing was rescued by D‐serine administration (Figure 6). Together, these findings support a model in which astrocytic TRPA1‐dependent D‐serine release enhances neuronal NMDA receptor signaling during prolonged sensory stimulation, thereby helping sustain neuronal activity and the associated CBV response.

In contrast, under resting conditions, astrocyte‐derived D‐serine contributes to the regulation of basal vascular tone by promoting vasodilation. We provide compelling evidence that astrocytes control vascular tone through BEST1/D‐serine signaling in the resting state in S1FL independent of sensory stimulation (Figure 8). Furthermore, we have demonstrated that direct infusion of D‐serine in astrocytes progressively dilates the cannulated arterioles ex vivo, which was blocked by KA, a GluN1 subunit‐specific NMDAR antagonist, even to the level below the baseline of vascular tone. D‐serine continued to induce robust arteriole dilation in the presence of TTX (Figure S11), indicating that this vascular response does not require action potential‐dependent neuronal activity. These findings are consistent with previous studies showing that astrocyte‐dependent and D‐serine‐induced vasodilation persist after neuronal activity is blocked and depend on endothelial GluN1 and eNOS signaling [35, 36, 37]. Thus, although our experiments do not directly identify the NMDA receptor‐expressing vascular cell type, our TTX and pharmacological results, together with previous vascular studies, support endothelial NMDA receptors as potential mediators of the resting‐state vasodilatory effect. These exciting possibilities await future investigations.

One of the limitations of the present study is that most experiments were performed under urethane anesthesia. Urethane was selected because it provides stable, long‐lasting anesthesia with relatively limited disruption of vascular physiology compared with several other commonly used anesthetics [40, 41, 65, 66]. Nevertheless, anesthesia could influence the magnitude and dynamics of sensory‐evoked neurovascular responses. To address this concern, we performed additional control experiments to test whether anesthesia depth could account for the altered CBV kinetics observed after astrocytic TRPA1 silencing (Figure S8). Using whisker stimulation under different concentrations of isoflurane anesthesia, we found that anesthesia depth mainly altered CBV response amplitude while largely preserving its temporal profile. In contrast, TRPA1 silencing shifted the response toward an earlier peak and selectively attenuated its late phase, indicating that the observed kinetic changes are unlikely to result solely from anesthesia depth. Although these controls reduce concerns regarding anesthesia‐related confounds, future studies in awake mice will be necessary to fully establish the physiological relevance of the astrocytic AQP4/TRPA1/BEST1/D‐serine pathway during sensory processing.

Additionally, the implantation of an acute window is another limitation to consider. Although our data showed that the acute window surgery did not induce gliosis (Figure S2), future experiments conducted with a chronic window would provide a more stable and long‐term observation of astrocytic activity without the confounding factors associated with acute surgical procedures. As the region of interest in this study was the S1FL, we employed the von Frey test to assess sensory thresholds to forepaw stimulation in vivo. It should be noted that the von Frey test is primarily designed to measure the immediate paw withdrawal response to filament application in mice, making it technically challenging to evaluate sensory responses to stimuli of different durations. A behavioral paradigm that can assess sensory responses to varying stimulation durations would better facilitate investigation of the AQP4/TRPA1/ BEST1/D‐serine signaling pathway in vivo in future studies.

In conclusion, our findings position astrocytic AQP4/TRPA1/BEST1/D‐serine signaling as a novel integrated molecular mechanism that prevents the progressive decay of neuronal and vascular responses when cortical activity must be maintained over time. Moreover, we identify astrocytic D‐serine as a regulator of basal vascular tone, highlighting the role of astrocytes in shaping resting neurovascular states. Taken together, these results propose astrocytes as key integrators that support prolonged cortical processing and vascular stability. Given that dysregulation of astrocytic AQP4 and associated signaling pathways has been implicated in neurological conditions such as Alzheimer's disease (AD) [67, 68], stroke [69], and traumatic brain injury (TBI) [70], our findings offer mechanistic insights that may inform future studies of neurovascular dysfunction in both physiological and pathological contexts.

## 4 Materials and Methods

### 4.1 Animals

Male C57BL/6J, C57BL/6N, TRPA1 KO, and TRPA1 floxed mice were used. C57BL/6J mice were purchased from RaonBio (Yongin, Republic of Korea), C57BL/6N mice were purchased from OrientBio (Seongnam, Republic of Korea), and were acclimated for at least 1 week before being used in this study. To generate TRPA1 KO, B6;129P‐*Trpa1^tm1Kykw^*/J (RRID: IMSR_JAX:006401) mice were purchased from Jackson Laboratory and backcrossed with C57BL/6J mice (RRID: IMSR_JAX:000664) for more than 10 generations and were considered congenic C57BL/6J mice. TRPA1 floxed mice were purchased from Jackson Laboratory (B6.129S‐*Trpa1^tm2Kykw^*/J, RRID: IMSR_JAX:008650). All animals were housed in a 12‐h light/12‐h dark cycle in a specific‐pathogen‐free facility with controlled temperature and humidity and had free access to food and water. All experimental procedures were conducted according to protocols approved by the Institutional Animal Care and Use Committee of IBS (IBS‐2022‐026), Sungkyunkwan University (SKKUIACUC2021‐04‐27‐1), and the SKKU Institutional Biosafety Committee (SKKUIBC2021‐04‐10‐1).

### 4.2 Virus Injection

Eight‐week‐old mice were anesthetized with 1–1.5% isoflurane throughout the surgery, following a brief induction of 3% isoflurane using an isoflurane vaporizer (VetEquip Inc., USA). To maintain the body temperature at 37°C, the mouse was placed on a temperature‐controlled heating pad (FHC Inc., USA). A small piece of skin was incised to expose the skull, and a small 1 mm diameter hole was drilled at coordinates 0 mm posterior and +1.3 mm lateral to bregma. The virus was injected stereotaxically into the hole at a 40° angle, targeting the forelimb region of the right primary somatosensory cortex (right S1FL). To silence the AQP4 gene, AAV‐pSicoR‐*Aqp4* shRNA‐mCherry [10, 39] (titer: 4.28 × 10^12^ GC/mL) diluted 1:1 in phosphate‐buffered saline (PBS) was used. As a control, AAV‐pSicoR‐scrambled shRNA‐mCherry (titer: 5.09 × 10^12^ GC/mL) was used. In the case of astrocyte‐specific *Trpa1* gene silencing, a 1:1 mixture of AAV‐GFAP‐mCherry‐Cre (titer: 2.49 × 10^12^ GC/mL) and AAV‐pSico‐TRPA1‐GFP [10] (titer: 2.97 × 10^12^ GC/mL) was used. As a control, AAV‐pSico‐scrambled shRNA‐GFP (titer 4.23 × 10^12^ GC/mL) was mixed instead of AAV‐pSico‐TRPA1‐GFP. For simultaneous two‐photon imaging of astrocytic Ca^2^^+^ signals and vascular dynamics following astrocyte‐specific TRPA1 knockdown, a mixture of three AAV vectors was injected. The mixture contained either AAV‐pSico‐scrambled shRNA (2.54 × 10^13^ GC/mL) as the control or AAV‐pSico‐*Trpa1* shRNA (1.37 × 10^13^ GC/mL) for TRPA1 knockdown, together with AAV‐GFAP‐Cre (2 × 10^12^ GC/mL) and AAV‐gfaABC1D‐Lck‐GCaMP6f (8.78 × 10^12^ GC/mL), which expressed the fluorescent Ca^2+^ indicator. The AAV‐pSico‐scrambled shRNA and AAV‐pSico‐*Trpa1* shRNA stocks were each diluted to approximately 3 × 10^12^ GC/mL before injection. Reporter‐free versions of the shRNA and GFAP‐Cre vectors were used to prevent spectral interference with GCaMP6f and the fluorescent vascular tracer during simultaneous imaging. All viruses were produced by the virus facility of IBS. The virus was loaded into a sharp glass pipette with a diameter of 40 µm and delivered in volumes of 1–1.4 µL at a rate of 80 nL/min using a 10‐µL Hamilton syringe (The Hamilton Company, USA) and a syringe pump (Harvard Apparatus, USA). The glass pipette was kept in place for at least 5 min after the injection to minimize backflow. After viral injection was complete, the drilled hole was filled with biocompatible silicone (KWIK‐SIL, World Precision Instruments, USA), and the skin was closed with surgical sutures (B. Braun Surgical S.A., Spain). The stereotaxic coordinates of the injection site for electrophysiology and behavior experiments were 2.25 mm lateral and 1.4 mm ventral to the bregma, injected bilaterally at a 90° angle. The animals were then returned to their home cages for a recovery period of at least 2 weeks before conducting the planned experiments.

### 4.3 Cranial Window Surgery for In Vivo Imaging Experiments

More than 2 weeks after the injection, an acute cranial window was implanted over the right S1FL region to conduct hemoglobin‐based intrinsic optical signal (IOS) imaging and two‐photon imaging. During surgical procedures, mice were anesthetized with 1–1.5% isoflurane, and body temperature was maintained at 37°C. A craniotomy was carefully conducted on the right S1FL region using a dental drill (Microtorque II, Ram Products Inc., USA), avoiding the viral injection site. The dura mater remained intact, and the hydrated exposed cortex was treated with either HEPES‐buffered saline (HBS; containing 135 mm NaCl, 5 mm KCl, 10 mm HEPES, 10 mm glucose, 2 mm CaCl_2_, 2 mm MgSO_4_) or HBS‐soaked GelFoam sponges (MS0005, Ethicon Inc., USA). After confirming the absence of microbleeding, a glass coverslip (4 mm, Deckglas, Germany) was placed over the exposed cortex. To ensure stability during imaging experiments, a metal holding frame was securely attached to the skull using glue, and a dental resin wall was built around the craniotomy site. Once all the surgical procedures were completed, the administration of isoflurane anesthesia was discontinued, and the mice were immediately switched to urethane anesthesia (1.25 g/kg, i.p.) for the in vivo imaging experiments. Heart rate and peripheral oxygen saturation (SpO_2_) were continuously monitored using a physiological monitoring system (PhysioSuite, Kent Scientific Corp., USA).

### 4.4 Intrinsic Optical Signal (IOS) Imaging and Analysis

The optical imaging system (Imager 3001‐Celox, Optical Imaging Ltd., Rehovot, Israel) was used to record the hemodynamic response of the right S1FL region following the forepaw electrical stimulation. Silicone oil (Sigma‐Aldrich, MO, USA) was used to fill the dental resin wall built around the S1FL region, which was then illuminated by an LED lamp (CLS150, Leica Microsystems CMS GmbH, Mannheim, Germany). Images were acquired as previously described [38]. The electrical stimulation was delivered to the left forepaw of mice under urethane anesthesia. The stimulation consisted of 500 µs electrical pulses at a frequency of 4 Hz and an amplitude of 0.5 mA, applied for a duration of 5 or 20 s using a pulse stimulator (Master‐9, A.M.P.I., Jerusalem, Israel) and a current generator (ISO‐Flex, A.M.P.I., Jerusalem, Israel). For experiments assessing the effects of anesthesia depth, hemodynamic responses in the barrel cortex were evoked by 50‐ms air‐puff pulses delivered at 5 Hz and a pressure of 25 psi using a Picospritzer III (General Valve Corp., Fairfield, CT, USA) under graded isoflurane anesthesia (0.5%, 1.0%, and 1.5%).

Raw imaging data from individual trials were imported from the BLK files and converted into 3D image stacks consisting of two spatial dimensions and time. Image stacks from all selected trials obtained under the same experimental condition were summed on a frame‐by‐frame and pixel‐by‐pixel basis and divided by the total number of trials to generate a trial‐averaged image stack.

For each pixel, the baseline intensity (I_0_) was calculated as the mean reflected‐light intensity during the 5 s immediately preceding stimulation onset. The signal at each subsequent time point was normalized to its pixel‐specific baseline according to the following equation: ΔI/I_0_(%) = − [(I(t)−I_0_)/I_0_] × 100, where I(t) represents the reflected‐light intensity at a given time point. Because an increase in cortical blood volume and total hemoglobin reduces reflected‐light intensity at this wavelength, the fractional intensity change was multiplied by −1 so that positive values represented an increase in the hemodynamic response. This normalization was independently applied to every pixel and every imaging frame to generate a spatiotemporal map of the percentage change in reflected‐light intensity. A region of interest (ROI) of 21 × 21 pixels was selected. The time series data were temporally averaged for each time point, and the peak amplitude was determined within the stimulation period. The onset time was determined as the time point when the intensity change exceeded the baseline mean plus 2 standard deviations.

For ex vivo IOS imaging, coronal brain slices were transilluminated using a controlled infrared (IR) light source with an optical filter (775 nm wavelength, Omega Filters), and images were taken using a microscope (Olympus, BX50WI) equipped with a digital CCD camera (Hamamatsu, ORCA‐R2). The relative change of transmittance (ΔT/T) was normalized to baseline (average of 5 images). Decay of the intrinsic optical signal (IOS) was measured by averaging the last 10 s of the response after dividing responses by the peak response. Imaging Workbench software (INDEC BioSystems) was used for image acquisition and analysis.

### 4.5 Local Field Potential (LFP) Recording and Analysis

The IOS system was utilized to identify the activated area within the right S1FL region, and the insertion site for the electrophysiology electrode was determined accordingly. An electrode with an impedance of approximately 0.5 MΩ (FHC, Inc., ME, USA) was positioned at the center of the activated area in the right S1FL region, specifically targeting Layer II/III of the somatosensory cortex at a depth of 300 µm. A screw was inserted into the skull above the left olfactory bulb region and connected to the ground wire to minimize electrical noise and interference. Electrophysiological activity during forepaw stimulation was recorded at a frequency of 40 kHz (Plexon Inc., TX, USA), and the raw electrophysiology data were filtered between 0.5 and 200 Hz for LFP analysis. The frequency range from 55 to 65 Hz was excluded from all analyses due to the presence of a notch filter set at 60 Hz. The analysis of LFP data was conducted using the Chronux toolbox, an open‐source software. The continuous LFP signal was aligned and averaged across repeated trials within each animal. The 5‐s interval immediately preceding stimulation onset was used as the pre‐stimulus baseline. Trials containing prominent motion artifacts, amplifier saturation, or unstable baseline activity were excluded before averaging.

To quantify the LFP response evoked by individual forepaw pulses, the continuous LFP recording was divided into consecutive 250‐ms segments, corresponding to the interval between stimuli delivered at 4 Hz. Each segment was aligned to the onset of the corresponding electrical pulse. Thus, 20 pulse‐aligned segments were extracted from each 5‐s stimulation trial, whereas 80 segments were extracted from each 20‐s stimulation trial. The pulse‐aligned segments were averaged within each trial and subsequently averaged across repeated trials to generate one representative stimulus‐triggered LFP waveform for each animal. The evoked LFP peak amplitude was defined as the magnitude of the largest negative deflection in the pulse‐triggered average waveform during the response period. The baseline neural activity value was obtained from the 5‐s pre‐stimulus period and calculated from the spectral power density within the analyzed LFP frequency range.

### 4.6 In Vivo Two‐Photon Imaging

Two‐photon imaging data were acquired using a 25× objective lens (N.A. 0.95, Leica Microsystems) in a two‐photon microscope (TCS SP8MP, Leica Microsystems, Germany) equipped with a Ti: Sapphire femtosecond laser source (Chameleon Vision II, Coherent, Inc., USA). The cortical vasculature was visualized by injecting Fluorescein isothiocyanate‐dextran (FITC, MW 70 kDa, 5% diluted in PBS) or Texas Red‐conjugated dextran (MW 70 kDa, neutral, 5% diluted in PBS) via the retro‐orbital sinus. To measure the changes in diameter in different compartments of the cerebral blood vessels following sensory stimulation, the two‐photon laser was excited at 820 or 910 nm. In the case of calcium imaging of astrocytes, the laser was excited at 910 nm. A bandpass filter (520 ± 50 or 624 ± 40 nm) was used to acquire the corresponding emitted fluorescent signals. The forepaw electrical stimulation was delivered as described above, and the two‐photon images were acquired at a rate of 5 Hz with a resolution of 512 × 512 pixels. All imaging data were processed using Fiji (ImageJ) and custom‐written MATLAB code (MathWorks, USA).

### 4.7 Calcium and Vessel Image Analysis

For astrocytic Ca^2+^ analysis, motion‐corrected images were spatially smoothed with a Gaussian filter in Fiji. The mean fluorescence intensity within each ROI was extracted from every imaging frame to generate fluorescence time courses. Calcium signals were expressed as ΔF/F_0_ using the following equation: ΔF/F_o_ = [F(t) − F_o_]/F_o_, where F(t) represents the fluorescence intensity at time t and F_0_ represents the mean fluorescence intensity during the 10‐s baseline period preceding forepaw stimulation. Sensory‐evoked astrocytic Ca^2+^ responses were quantified by calculating the peak ΔF/F_0_ amplitude and the area under the curve (AUC).

For vascular analysis, pial arteries and penetrating arterioles were identified based on their anatomical location and morphology. Pial arteries were identified as horizontally oriented vessels running along the cortical surface, whereas penetrating arterioles were identified as branches descending from the pial arterial network into the cortical parenchyma and appearing as circular or oval cross‐sections in the imaging plane. Image sequences containing individual pial arteries or penetrating arterioles were isolated from each imaging trial. The vascular images were processed using a Gaussian filter to improve the signal‐to‐noise ratio. For pial arteries, a fluorescence intensity profile was drawn perpendicular to the longitudinal axis of the vessel in each imaging frame, and the vessel diameter was calculated as the full width at half maximum (FWHM) of the intensity profile. For penetrating arterioles, horizontal and vertical fluorescence intensity profiles spanning the entire vessel cross‐section were generated in each frame. The FWHM was calculated for each profile, and the maximum FWHM value was taken as the arteriole diameter. These measurements were repeated across all frames to generate the temporal profile of vessel diameter for each trial. Changes in vessel diameter were normalized to the pre‐stimulation baseline and expressed as: ΔD/D_o_ × 100 (%) = [D(t) − D_o_]/D_o_ × 100, where D(t) represents the vessel diameter at time t and D_0_ represents the mean diameter measured during the 10‐s baseline period preceding forepaw stimulation. Vascular responses were quantified by determining the peak percentage change in diameter and the AUC of the diameter‐response trace.

For ex vivo astrocytic Ca^2+^ imaging, slices were transferred to a chamber perfused with carbogenated aCSF at 2–3 mL/min using a peristaltic pump (Miniplus 3, Gilson). Ca^2+^ signals were acquired on an A1R MP^+^ confocal microscope (Nikon). Ca^2+^ images were obtained using a krypton/argon laser (488 nm excitation and >495 nm emission) and acquired at 2 frames/s with a resolution of 256 × 256 pixels for ∼90s.  For the evoked astrocytic Ca^2+^ responses, the tungsten bipolar electrode was placed in cortical Layer 5. Astrocytic Ca^2+^ responses in cortical layer II/III were evoked by 10 Hz stimulation (100 µA, 0.1‐ms pulse duration for 5 or 20‐s) via a constant current isolation unit (A365, World Precision Instruments). Ca^2+^ transients were calculated as ΔF/F_0_  =  (F − F_0_)/(F_0_ − F_b_), where F_0_ is the baseline fluorescence measured before quinpirole application (Base condition), and Fb is the background fluorescence defined from a manually selected background ROI.

### 4.8 Habituation to Handling and Experimental Environment for Behavioral Tests

All mice were gently handled daily for 3 days before the first day of experiments. Mice were given at least 1 h to habituate in the behavioral room before experiments.

### 4.9 Open Field Test

Mice were placed into an open field chamber (40 cm × 40 cm × 40 cm) and allowed to freely explore the area for 10 min. The center was defined as an area in the middle of the chamber (20 cm × 20 cm). An automated tracking system (EthoVision) was used to monitor and analyze the animal's locomotion.

### 4.10 Von Frey Filament Test

The test was performed between 1:00 pm–6 pm in an isolated room maintained at 22 ± 2°C and 50 ± 10% humidity. For the mechanical threshold (von Frey filament) testing, mice were brought from the standard animal housing room and placed in a transparent plastic cylinder (7 cm diameter and 20 cm height) on a metal mesh floor with 5 × 5 mm holes (mesh floor, Jeung Do Bio & Plant Co.). The mice were then habituated for at least 2 h prior to the test. To assess mechanical sensitivity, the withdrawal threshold of the left forepaw was measured using a series of von Frey filaments (touch test sensory evaluation, kit of 20, Cat. No 58011, Stoelting co.) (2.44, 2.83, 3.22, 3.61, 3.84, 4.08, 4.17, 4.31 nm, Exacta, North Coast Medical, USA; equivalent in grams to 0.04, 0.07, 0.16, 0.4, 0.6, 1, 1.4, 2). The 50% withdrawal threshold was determined using the ‘up‐down’ method, which has been previously described [71]. The withdrawal response to the filaments was regarded as a forepaw lift or flinch. The 0.4 g filament was the first stimulus to be used. When there was no response, the next higher‐force filament was applied, and when there was a response, the next lower filament was applied. A total of 5 trials, beginning from the first positive response, were measured. For the D‐serine rescue experiment, D‐serine 600 mg/kg in saline was intraperitoneally injected before placing in the cylinder. The von Frey withdrawal threshold was calculated as the 50% mechanical withdrawal threshold (g), based on previous statistical techniques [71] as follows:

$$50\%\;threhold\;\left(g\right)=10^{X_{f}+\kappa \delta }/10,000$$

X_f_ = Value of the Final von Frey Filament Used in Log Units

κ = tabular value (for the pattern of responses)

δ = mean difference between stimuli in log units

### 4.11 Heterologous Expression in HEK293T Cell Lines

Human embryonic kidney (HEK) 293T cells were purchased from ATCC (CRL‐3216). The cell line has been tested for mycoplasma contamination. HEK293T cells were cultured in DMEM (10‐013, Corning) supplemented with 10% heat‐inactivated fetal bovine serum (10082‐147, Gibco) and 10 000 units/mL penicillin‐streptomycin (15140‐122, Gibco) at 37°C in a humidified atmosphere of 95% air and 5% CO_2_. According to the manufacturer's protocol, the transfection of expression vectors was performed with Effectene Transfection Reagent (Effectene, 301425, Qiagen). One day before performing the experiments, HEK293T cells were transfected with each DNA (CMV10‐3xFLAG‐TRPA1 and pRK5‐HA‐AQP4), 1 µg per 35 mm dish.

### 4.12 Co‐Immunoprecipitation and Western Blot Assay

Equal amounts of protein lysates prepared with IP lysis buffer (Thermo Scientific, #87787) were incubated with 25 microliters of ANTI‐FLAG M2 Affinity Agarose Gel (A2220, Sigma) overnight. The beads were washed five times with IP buffer. Bound proteins were eluted from beads with SDS/PAGE sample buffer followed by immunoblotting. Blot analyses were performed with antibodies for HA (1:500, Purified anti‐HA.11 Epitope Tag Antibody, MMS‐101P, BioLegend) and FLAG (1:500, Monoclonal anti‐FLAG M2 antibody, F1804, Sigma).

### 4.13 Brain Slice Preparation for In Situ Experiments

Mice were anesthetized with 3% isoflurane, and transcardiac perfusion was performed with saline followed by 4% paraformaldehyde (PFA). Isolated brains were postfixed in 4% PFA at 4°C for 24 h followed by 30% sucrose solution at 4°C for 48 h. The fixed brains were frozen, embedded in optimal cutting temperature (OCT) compound (FSC 22 Frozen Section Media, Leica, IL, USA), and cut into 30 µm coronal sections with a cryostat microtome (CM1950, Leica, IL, USA).

### 4.14 In Situ Proximity Ligation Assay

Duolink In Situ Red Starter Kit Mouse/Rabbit (DUO92101, Sigma‐Aldrich) was used. On the day of the experiment, coronal brain tissues containing the S1FL were permeabilized in 0.3% Triton‐X containing PBS. The tissue sections were incubated in blocking solution for 60 min at 37°C. After blocking, the samples were incubated with rabbit anti‐TRPA1 (1:100, ab62053, Abcam) and mouse anti‐AQP4 (1:100, ab9512, Abcam) overnight at 4°C. The following day, samples were incubated with anti‐rabbit MINUS and anti‐mouse PLUS probes, ligase, and polymerase sequentially. DNA strands participate in rolling circle DNA synthesis only when the two probes are in close proximity (<40 nm). Fluorescent‐labeled complementary oligonucleotide probes were observed under Zeiss confocal microscopy.

### 4.15 Immunohistochemistry

Frozen coronal sections (40 µm thick) were prepared as previously described [72] and were blocked in a solution containing 10% donkey serum in universal blocking solution (00‐8120, Invitrogen, USA) for 1 h at room temperature. Following this, the sections were incubated overnight at 4°C with primary antibodies in PBS, and then washed three times for 5 min each with PBS. Subsequently, the sections were incubated with secondary antibodies for 2 h at room temperature, followed by another three washes of 5 min each with PBS. Nuclear counterstaining was performed using a 100 ng/ml 4,6‐diamidino‐2‐phenylindole (DAPI) solution (1:10000) in PBS for 10 min. The primary antibodies used were rabbit anti‐AQP4 antibodies (1:800, AB2218, Millipore), rabbit anti‐TRPA1 (1:100, ab62053, Abcam), mouse anti‐GFAP (1:400, MAB3402, Millipore), rabbit anti‐cFos (1:400, ab222699, Abcam), mouse anti‐NeuN (1:400, ab104224, Abcam) and the signals were visualized using secondary antibodies conjugated with Alexa Fluor 488 or Alexa Fluor 568 (1:350, Invitrogen). Fluorescence images were acquired using Zeiss confocal microscopy (63×) or a TCS SP8 confocal microscope (Leica Microsystems) (20×). The obtained images were analyzed using ImageJ and Imaris (Bitplane, UK, RRID: SCR_007370) software.

### 4.16 Brain Slice Preparation for Electrophysiology

Mice were anaesthetized with isoflurane and decapitated to isolate the brain. Coronal slices (300 µm thick) containing the somatosensory cortex were cut with a vibratome (DSK Linear Slicer, Kyoto, Japan). Briefly, slices were cut in ice‐cold dissection buffer containing 5 KCl, 1.25 NaH_2_PO_4_, 26 NaHCO_3_, 10 D‐glucose, 0.5 CaCl_2_, 10 MgCl_2,_ and 212.5 sucrose (in mm), bubbled with 95% O_2_/ 5% CO_2_ (pH 7.4). Slices were transferred to standard artificial cerebrospinal fluid (aCSF) containing 130 NaCl, 24 NaHCO_3_, 1.25 NaH_2_PO_4_, 3.5 KCl, 1.5 CaCl_2_, 1.5 MgCl_2_, and 10 sucrose (in mm), bubbled with 95% O_2_/ 5% CO_2_ (pH 7.4) and incubated at room temperature to recover for at least 1 h before recording.

### 4.17 Whole‐Cell Patch Clamp Recordings

Whole‐cell recordings of pyramidal neurons in the somatosensory cortex were acquired in acute coronal brain slices. Borosilicate glass pipettes (outer diameter, 1.5 mm; internal diameter, 0.86 mm; 1B150F‐4, World Precision Instruments) with resistances ranging from 5 to 8 MΩ were pulled using a laser micropipette puller (PC‐100, Narishige). The pipette was filled with an internal solution containing 120 CsMeSO_4_, 5 NaCl, 4 CsCl, 10 HEPES, 5 EGTA, 4 Mg‐ATP, 0.3 Na_2_‐GTP, and 5 QX‐314 (in mm), pH adjusted to 7.3 with CsOH (278–285 mOsmol). Whole‐cell voltage‐clamp recordings were performed using a MultiClamp 700B amplifier (Molecular Devices), filtered at 2 kHz, and digitized at 10 kHz using a Digidata 1550B digitizer (Molecular Devices). Tonic NMDAR current recordings were performed as previously described [30]. Briefly, baseline current was stabilized under treatment of CNQX (20 µm), Bicuculline (10 µm), CGP 55845 (10 µm), and Strychnine (10 µm). Delta D‐serine (ΔD‐serine) level was measured by an increase in baseline shift after D‐serine application (100 µm), and the amplitude of I_tonicNMDAR_ was measured by a decrease in baseline shift after APV application (50 µm) in voltage holding at +40 mV. Tonic NMDAR GMS occupancy was measured as follows:

$$\mathrm{Tonic}\;\mathrm{NMDAR}\;\mathrm{GMS}\;\mathrm{occupancy}=\frac{I_{tonicNMDAR}}{I_{tonicNMDAR}+\Delta D-\mathrm{serine}}$$

### 4.18 Ex Vivo Parenchymal Arteriole Cannulation

PA (parenchymal arteriole) was visualized using a 40x Nikon objective (NIR Apo, 40/0.8w, DIC N2, ∞/0 WD 3.5) equipped with infrared differential interference contrast (IR‐DIC) optics. Cannulas (inner diameter, 1.17 mm; outer diameter, 1.50 mm; G150TF‐3, Warner Instruments) were pulled with a micropipette puller (P‐97 puller, Sutter Instruments) and mounted onto a micromanipulator. Luminal flow was controlled using a pressure ejection system (PDES ‐02DX, npi electronic GmbH). A pressure transducer was placed just before the cannula for constant pressure monitoring (servo pump; PS/200, Living System Instrumentation), as previously described [73]. The internal cannula solution consisted of the following: 3 KCl, 135 NaCl, 1 MgCl_2_, 10 glucose, 10 HEPES, 2 CaCl_2_ (in mm), and 1% albumin with osmolarity at 300–305 mOsm and pH 7.4 adjusted with NaOH. To establish comparable basal vascular tone between groups (Figure 8), the cannulated arterioles were pressurized at a low‐pressure value of ∼15 mmHg, and then further constricted by bath application of U46619 (Thromboxane A2 receptor agonist, 100 nm) [53]. Changes in vascular tone were measured before and after D‐serine (20 mm) infusion via astrocytic syncytium in the absence and presence of kynurenic acid (KA, 1 mm). To prevent neuronal effects on vascular diameter, TTX (1 µm) was bath‐applied during experiments (Figure S11). Diameter values are expressed as % diameter to the basal level of vascular tone.

### 4.19 D‐Serine Loading Through Astrocytic Gap‐Junction‐Coupled Syncytium

Cortical astrocytes (within cortical layers II/III) in close proximity (∼200 µm) to a pyramidal neuron or PA [74] were identified with a 40× water‐immersion objective. Patch pipettes were made from thin‐walled borosilicate glass (outer diameter, 1.5 mm; internal diameter, 0.86 mm; 1B150F‐4, World Precision Instruments) and pulled (PC‐100, Narishige) to resistances between 8 and 10 MΩ. The internal solution in astrocytes consisted of the following: 80 K‐gluconate, 60 KCl, 10 HEPES, 5 EGTA, 2 Mg_2_ATP, 0.2 Na_2_GTP, 0.1 Alexa 488 with or without 20 D‐serine (in mm) (see Figures 4 and 6). The osmolarity was 285–289 mOsm, and pH was adjusted to 7.3 with KOH.

### 4.20 Statistics

Off‐line analysis was carried out using Clampfit version 10.6.0.13, GraphPad Prism version 9.3.0, and IBM SPSS Statistics (SPSS Inc., IL, USA, RRID:002865.). To assess the normality of the data, Shapiro‐Wilk tests or Kolmogorov‐Smirnov tests were conducted depending on the sample size. When comparing between two independent samples, statistical significance was assessed by Student's two‐tailed unpaired t‐test when samples showed a normal distribution and by the Mann‐Whitney test when samples did not pass the normality test. Samples that passed the normality test but not the equal variance test were assessed with Welch's correction. To assess the significance of paired data collected from the same mouse, we employed a paired‐samples t‐test. Comparisons among more than two groups were performed using one‐way ANOVA with Tukey's post‐hoc test when data passed the normality test and Kruskal‐Wallis test with Dunn's post‐hoc test when data did not pass the normality test. Significance levels were given as: n.s. *p* > 0.05, ^*^ *p* < 0.05, ^**^ *p* < 0.01, ^***^ *p* < 0.001 and ^****^ *p* < 0.0001. All data are presented as the mean ± standard error of the mean (SEM).

Illustrations Were Created With BioRender.com.

## Author Contributions

Conceptualization: K.H, M.W.J, T.P, K.J.K., M.S., and C.J.L. Methodology: K.H, M.W.J, T.P, K.J.K., M.S., and C.J.L. Validation: M.S., and C.J.L. Formal analysis: K.H, M.W.J, T.P, K.J.K. Investigation: K.H, M.W.J, T.P, K.J.K., S.K., T.Y.K, M.G.P, M.K., and S.W.C. Resources: M.S., and C.J.L. Writing: K.H, M.W.J, T.P, K.J.K., M.S., and C.J.L. Visualization: K.H, M.W.J, T.P, K.J.K. Supervision: M.S., and C.J.L. Project administration: M.S., and C.J.L. Funding acquisition: K.H, M.S., and C.J.L.

## Ethical Statement

All experimental procedures were conducted according to protocols approved by the Institutional Animal Care and Use Committee of IBS (**IBS‐2022‐026**), Sungkyunkwan University (**SKKUIACUC2021‐04‐27‐1**), and the SKKU Institutional Biosafety Committee (**SKKUIBC2021‐04‐10‐1**).

## Conflicts of Interest

The authors declare no conflicts of interest.

## Supporting information




**Supporting File**: advs77878‐sup‐0001‐SuppMat.xlsx.

## Data Availability

The data that support the findings of this study are available from the corresponding author upon reasonable request.
